# Systematic optimization and characterization of bacterial depolymerization of poly(ethylene terephthalate) plastics

**DOI:** 10.1038/s41598-026-58899-9

**Published:** 2026-06-20

**Authors:** Apoorva Sherigar, Ritu Raval, Abdul Ajees Abdul Salam, Sudarshan Acharya, Chuxia Lin, Subbalaxmi Selvaraj

**Affiliations:** 1 Manipal Institute of Technology, Manipal Academy of Higher Education, Manipal, 576104 Karnataka India; 2 Manipal Institute of Applied Physics, Manipal Academy of Higher Education, Manipal, 576104 Karnataka India; 3 School of Engineering, Faculty of Science, Engineering and Built Environment, Deakin University, Burwood, VIC 3125 Australia

**Keywords:** Poly(ethylene terephthalate), *Glutamicibacter mysorens* ASR14, Response surface methodology, Sustainable plastic bioremediation, Analytical characterization, Biological techniques, Biotechnology, Environmental sciences, Microbiology

## Abstract

Biodepolymerization of poly(ethylene terephthalate) (PET) plastics using microorganisms has emerged as a promising and sustainable approach for mitigating pollution caused by PET waste. In this study, *Glutamicibacter mysorens* ASR14, a mesophilic bacterium isolated from Kodungaiyur dumpyard (Chennai, India), showed 27.6% PET biodepolymerization in terms of weight loss in 30 d. A customized screening of 20 trials was designed using JMP statistical software to evaluate the influence of various variables. Furthermore, a Central Composite Design (CCD) of Response Surface Methodology (RSM) was adopted and validated using four variables at five levels, with 25 trials, to correlate the relationship for enhanced PET biodepolymerization. A maximum PET weight loss of 75.6% was achieved in 60 d, representing a 2.73-fold improvement compared to that under unoptimized conditions. Enzymatic assays confirmed the involvement of esterase (5,690 U/mL) and lipase (962 U/mL) activities in accelerating the breakdown of PET. The analytical characterization techniques revealed significant surface erosion, reduction in crystallinity, and high yield of terephthalic acid (TPA), which also holds potential value for biorefinery applications. This work represents the first comprehensive report on process optimization for PET biodepolymerization using *G. mysorens* ASR14 as a whole-cell biocatalyst. The findings establish *G. mysorens* ASR14 as a promising candidate for developing scalable, green bioremediation strategies.

**Supplementary Information:**

The online version contains supplementary material available at https://doi.org/10.1038/s41598-026-58899-9.

## Introduction

Poly(ethylene terephthalate) (PET), a linear thermoplastic derived from crude oil, is one of the most widely used synthetic polymers globally. Composed of intermediates terephthalic acid (TPA) and ethylene glycol (EG), PET is renowned for its excellent properties, including high mechanical strength, chemical resistance, thermal stability, and capacity for producing high-quality products in various industrial sectors^1^. The growing use has resulted in a worldwide waste management dilemma, with over 240 million tonnes of PET waste being produced annually^2^. This has forced us to find ways to deal with the ever-increasing generation of PET waste^3^. Currently, approximately 60% of post-consumer PET is either incinerated or landfilled, with only 9% being recycled^4^. While recycling reduces plastic accumulation, the process can generate microplastics (MPs) through mechanical abrasion and material processing^5^. These microplastics further form nanoplastics (NPs) under environmental weathering conditions such as UV radiation, thermal stress and microbial activity, leading to accumulation in terrestrial and aquatic ecosystems^6^. Recent studies have highlighted the role of microplastics as vectors for heavy metals, which can enter the food chain, induce oxidative stress, inflammatory responses, cytotoxicity and endocrine disruption^7^.

In light of these challenges, biological recycling has emerged as an effective alternative for addressing the significant buildup of PET waste^8^. Over the past 15 years, there have been considerable advances in our understanding of the enzymatic breakdown of hydrolyzable polymers. Numerous PET-degrading enzymes from diverse microorganisms have previously been identified and characterized, including cutinases, lipases, esterases, carboxylesterases, PETases, and MHETases, which catalyze ester bond to produce soluble intermediates and monomers^9,10^. Recent studies have focused on enzymatic PET biodepolymerization involving isolation, purification, immobilization or engineering enzymes under controlled conditions^11,12^. Significant advances have been achieved through rational protein engineering, directed evolution and computational redesign. However, the purified enzyme systems show several limitations, such as reduced stability, substrate limitation, high costs associated with enzyme production, recovery and reuse^13^. Enzymatic depolymerization breaks down PET polymer, and complete assimilation is not achieved since intermediates Mono-(2-hydroxyethyl) terephthalate (MHET), Bis-(2-hydroxyethyl) terephthalate (BHET), and TPA tend to accumulate and inhibit PETase activity^14^. In contrast, the whole-cell microbial depolymerization approaches involve using microorganisms capable of colonizing the PET surface, secreting extracellular enzymes and assimilating the degradation intermediates^15^. It represents an environmentally relevant approach as microorganisms simultaneously produce hydrolytic enzymes, biosurfactants and possess transport systems that facilitate polymer depolymerization, intermediate uptake and intracellular metabolism without the need for any enzyme purification^16^. Nevertheless, the rate of whole-cell PET biodepolymerization remains relatively low under conventional conditions, highlighting the need for process optimization to improve microbial activity and enzyme production^17^. Whole-cell biodepolymerization is an efficient approach because microbial consortia or genetically engineered strains can collaboratively decompose PET into its constituent monomers^18^. Microorganisms play a pivotal role in PET bioremediation due to their prevalence in polluted environments. Extracellular enzymes produced by microbes break down complex molecules into simple, non-toxic metabolites that serve as nutrient sources to promote microbial growth^19^.

The efficiency of biodepolymerization depends on various environmental and nutritional factors, and understanding the variation of these variables is crucial for creating optimal conditions required for microorganisms to break down the substrate into target products^20^. Optimizing culture conditions is vital for an effective biotreatment process; hence, it is imperative to evaluate and identify the variables that exert the most substantial effect on enhancing biodepolymerization^21^. The conventional method of ‘one factor at a time’ (OFAT) is traditionally used for culture medium optimization^22–24^. However, when multiple variables are to be examined, this method is time-consuming, expensive and impractical. In contrast, statistical optimization provides a comprehensive understanding of the interactive effects of variables that impact the response of the system. Among the available statistical techniques, response surface analysis is essential for precisely identifying optimal conditions^25^.

Several studies have investigated PET biodegradation using purified enzymes, engineered microorganisms and microbial consortia, and reports on statistical optimization of whole-cell microplastic biodepolymerization remains limited. Previous optimization studies used Box-Behnken design (BBD) to assess the degradation through weight loss measurements or genomic evidence, whereas quantitative analysis of biodepolymerization products was not performed^26,27^. Limited information is available regarding the combined influence of multiple nutritional and physical variables involved in microplastic biodepolymerization using a single bacterial isolate. Therefore, this study aimed to screen, identify, characterize and report the bacterial isolate *G.mysorens* ASR 14 for PET biodepolymerization and develop a statistically optimized whole-cell biodegradation system through combined optimization of nutritional variables and physical variables customized specifically to the physiology of the bacteria, enabling evaluation of the interacting variables influencing PET biodepolymerization. By adjusting essential variables, this study aims to improve the efficiency of PET depolymerization and contribute to the advancement of sustainable plastic waste management strategies. The biodepolymerization efficiency and by-products were further assessed using various analytical techniques, such as gravimetric weight loss methods, scanning electron microscopy (SEM), Thin layer chromatography (TLC), Fourier transform Infrared spectroscopy (FTIR), High performance liquid chromatography (HPLC), Gas chromatography- mass spectroscopy (GC-MS), Raman spectroscopy, Nuclear magnetic resonance spectroscopy (NMR) and X-ray diffractometery (XRD). This work contributes to the development of an economically feasible strategy for PET microplastic bioremediation, supporting the Sustainable Development Goals (SDGs), particularly SDG 13 (Climate Action) by providing eco-friendly alternatives to emission-intensive treatment methods, SDG 14 (Life Below Water) and SDG 15 (Life on Land) by minimizing PET pollution in aquatic and terrestrial ecosystems.

## Materials and methods

PET powder (> 300 microns) was obtained from Zed Square Corporation (Santej, Gujarat, India). PET powders of particle size 150 μm, 90 μm, 75 μm, and 45 μm were separated using a mechanical sieve shaker (Aimil Ltd). TPA, p-nitrophenol acetate (p-NPA), and rhodamine B used in the experiment were obtained from HiMedia. The solvents, acetonitrile, toluene, methanol, formic acid, and TLC aluminum sheets of silica gel 60 F_254,_ were purchased from Merck, India. p-nitrophenol palmitate (p-NPP) was purchased from Sigma-Aldrich. All other chemicals used in the experiments were of analytical grade.

### Isolation and screening of PET-degrading microorganisms from dump yard soil

Soil samples were collected from Kodungaiyur dumping ground that spreads across 252 acres with 66.52 lakh tonnes of solid waste (coordinates 13°07’37.6” N and 80°16’48” E), Chennai, India. In the laboratory, one gram of fresh dump yard soil was added to a 100 mL Erlenmeyer flask containing 25 mL of sterilized Mineral Salt Media (MSM). 1% PET powder of particle sizes ranging from 45 to 300 μm was used for preliminary screening of bacterial isolates. The MSM composition in g/L was as follows: Na_2_HPO_4_−1.8 g, KH_2_PO_4_−0.9 g, NH_4_Cl-0.3 g, NaCl-0.15 g, MgSO_4_.7H_2_O-0.25 g, FeSO_4_.7H_2_O-0.01 g, CaCl_2_.2H_2_O-0.002 g, MnSO_4_.7H_2_O-0.001 g, CuSO_4_.5H_2_O-0.001 g, and ZnSO_4_.7H_2_O-0.001 g with the pH being set at 7.0 ± 0.2. The culture flasks were incubated for 7 d in an orbital shaker with the temperature and speed being set at 32 °C and 180 rpm, respectively. Serial dilutions were plated on 1% PET-MSM agar plates. The colonies exhibiting distinct morphologies were subsequently isolated and re-streaked on fresh 1% PET-MSM agar plates. The isolated bacterial colonies were subjected to three enrichment cycles in liquid MSM with 1% PET powder, incubated at 32 °C and 180 rpm for 90 d. All isolates from the enrichment culture were preserved on 1% PET-MSM agar plates, and periodic subculturing was performed to ensure viability.

### Screening of PET biodepolymerization in liquid media

Two loopful of bacterial isolates grown on 1% PET-MSM agar plates were inoculated in 25 mL of MSM and incubated at 32 °C for 16 h. Subsequently, 100 mL Erlenmeyer flasks containing 50 mL of MSM with 1% PET powder were inoculated with 4 mL of 16 h culture. The depolymerization of PET by the bacterial isolates was assessed by quantifying the release of TPA into the medium using a UV-Vis spectrophotometer (GENESYS 180 UV-Vis spectrophotometer; Thermo Scientific). TPA exhibits maximum absorbance in the 240–260 nm region owing to the presence of an aromatic ring^28^. A wavelength scan was performed to determine the maximum absorbance wavelength (λ_max_) of TPA in MSM (Fig. S1), and a series of TPA standards (0.1 mM-1 mM) were prepared in MSM to generate a calibration curve (Fig. S2). MSM was used as the blank during the spectrophotometric analysis to eliminate the interference from media components. The culture supernatants were collected and centrifuged at 10,000 rpm for 15 min (Eppendorf 5804 R refrigerated centrifuge), and the cell-free extract was analyzed to quantify the TPA concentration^29^.

### Biochemical characterization of bacterial isolate

The biochemical characterization of the bacterial isolate was performed using standard microbiological protocols to determine its phenotypic and physiological characteristics^30,31^.

#### Catalase test

Bacterial colony grown on 1% PET-MSM agar plate was transferred to a clean glass slide, to which 2 drops of 3% hydrogen peroxide was added. Formation of oxygen bubbles indicated a positive catalase reaction.

#### Methyl red (MR) test

The bacterial isolate was inoculated in glucose phosphate broth containing peptone (7 g/L), glucose (5 g/L) and dipotassium phosphate (5 g/L) and incubated at 37 °C for 24 h. 3–5 drops of methyl red reagent was added. The development of red colour indicated a positive result, whereas the yellow-orange colour indicated a negative result.

#### Voges–Proskauer (VP) test

The bacterium was incubated in glucose phosphate broth at 37 °C for 24 h. The culture broth was transferred to a sterile test tube, and 0.6 mL of α-naphthol reagent and 0.2 mL of 40% potassium hydroxide solution were added and allowed to stand for 15–30 min. The development of pink colour indicated a positive VP reaction.

#### Indole test

The bacterial isolate was grown in tryptone broth containing tryptophan and incubated at 37 ° C for 24–48 h. After incubation, 3–5 drops of Kovac’s reagent was added. The development of a red or pink coloured ring indicated a positive indole reaction.

#### Triple sugar iron (TSI) test

TSI agar medium (Himedia) was prepared and sterilized. The bacterium was inoculated into the TSI agar slant by stabbing and streaking the surface of the slant, and was incubated at 37 ° C for 24–48 h. After incubation, the reactions were interpreted based on colour changes and gas formation.

#### Starch hydrolysis assay

Starch agar medium containing soluble starch (10 g/L), peptone (5 g/L), yeast extract (3 g/L) and agar (15 g/L) was prepared with a pH of 7.0. The bacterial culture was inoculated and incubated at 37 ° C for 24–48 h. The plates were flooded with iodine solution. The appearance of clear hallow zone in the dark blue-black background indicated starch hydrolysis.

### Qualitative and quantitative assessment of enzymatic activity

#### Qualitative agar plate assays

Esterase activity was evaluated using p-NPA as a substrate in nutrient agar medium. 1 mL of a 50 mM solution of p-NPA in ethanol and 500 µL of phenol red were filter-sterilized, mixed into a sterilized and cooled nutrient agar medium, and plated under aseptic conditions. The crude enzyme was added to the agar wells and incubated for 24–48 h at 37 °C for colour change^32^. Lipase activity was determined using olive oil as a substrate in nutrient agar. Olive oil was emulsified with 0.2% Tween 60 and added to a sterilized medium with 0.01% rhodamine dye. Crude enzyme extract was added to the wells and incubated at 37 °C for 72 h. The agar plates were visualized under a UV lamp at 350 nm for colour change^33^. Protease activity was assessed using skim milk agar. Sterilized media were inoculated with the bacterial culture and incubated for 48 h to observe halo zone formation.

#### Quantitative enzyme assays

Esterase activity was quantified by analyzing the absorbance change due to the hydrolysis of p-NPA (100 mM) dissolved in DMSO, using 50 mM phosphate buffer at pH 7.4^34^. Lipase activity was assessed using p-NPP as the substrate. The cell-free supernatant was added to a substrate mixture containing 15 mg of p-NPP in 5 mL isopropanol and 25 mg of gum arabic in 25 mL 50 mM phosphate buffer (pH 7.4). Absorbance for esterase and lipase activity was measured spectrophotometrically at 402 nm after 30 min incubation at 37°C^35^. Protease activity was determined according to the protocol described by Jayashree et al.^36^ The enzyme solution was added to a 0.5% (w/v) casein solution (dissolved in 50 mM citrate buffer, pH 7.6) and incubated at 37 °C for 60 min. The reaction was terminated by adding 1 mL of 10% (w/v) trichloroacetic acid and centrifuged at 10,000 rpm for 10 min. 0.5 mL supernatant was mixed with 0.5 M Na_2_CO_3_ and Folin–Ciocalteu’s reagent: water (1:2 v/v) and incubated at room temperature for 30 min in the dark.

### Identification of bacterial isolate by 16 S rRNA sequencing

The bacterial isolate was outsourced to MACS Centre for Microorganisms at Agharkar Research Institute in Pune, India, for 16 S rRNA sequencing. Total genomic DNA was extracted using a GeneElute Genomic DNA isolation kit (Sigma, USA) following the manufacturer’s guidelines. PCR was performed using 27 F and 1492R primers to amplify the 16 S rRNA gene^37^. PCR was carried out using the Eppendorf Gradient Master cycler system with a cycle of initial denaturation at 94 °C for 3 min, 32 cycles of denaturation at 94 °C for 45 s, annealing at 51 °C for 1 min, extension at 72 °C for 90 s, and final extension at 72 °C for 10 min, after which the mixture was held at 4 °C. A Magnetic bead-based method was used to clean the PCR products. BigDye™ Terminator v3.1 Cycle Sequencing Kit (Applied Biosystems) was used to sequence the PCR products. Forward and reverse primers were used to sequence nearly complete 16 S rRNA gene. The products after cycle sequencing were cleaned using BigDye XTerminator™ Purification Kit (Applied Biosystems). The samples were analyzed using a SeqStudio 232,002,103 (Applied Biosystems). Sequencing data were processed using DNA Analyzer computer software version 1.1.4. The 16 S rRNA gene sequences were analyzed using EzTaxon. A phylogenetic tree was constructed based on a bootstrap test using the neighbor-joining algorithm in MEGA 11 software^38^.

### Shake flask culture optimization of PET biodepolymerization

A customized screening of variables was performed using JMP statistical software to screen and evaluate the critical variables. The variables were selected based on their ability to influence the biodepolymerization process, such as microbial growth, enzymatic activity, substrate accessibility, and metabolic adaptation. Twenty experimental trials were performed to determine the effects of 10 variables (six categorical and four continuous variables). The variables evaluated were carbon sources (X_1_) (at 1% w/v: glucose (X_1_L1), sucrose (X_1_L2), lactose (X_1_L3), xylose (X_1_L4), and fructose (X_1_L5)), nitrogen sources (X_2_) (at 1% w/v: yeast extract (X_2_L1), peptone (X_2_L2), casein (X_2_L3) and soybean meal (X_2_L4)), surfactants (X_3_) (at 0.1% v/v: Tween 20 (X_3_L1), Triton X-100 (X_3_L2), and sodium dodecyl sulfate (SDS) (X_3_L3)), basal media (X_4_) (MSM (X_4_L1) and Bushnell Haas Broth (BHB) (X_4_L1)), amino acids (X_5_) (at 0.1% w/v: No amino acid (X_5_L1), leucine (X_5_L2), glutamine (X_5_L3), and cysteine (X_5_L4)), PET particle size (X_6_) (45 μm (X_6_L1), 90 μm (X_6_L2), and 150 μm (X_6_L3)), PET percentage (X_7_) (at 1% w/v and 5% w/v), inoculum size (X_8_) at 5% v/v and 25% v/v, agitation speed (X_9_) at 120 rpm and 200 rpm, and pH (X_10_) at 5 and 7. The incubation temperature was maintained at 32 °C. The list of variables used in the customized screening design is provided in Table 1, and the design matrix of trials with experimental and predicted responses is depicted in Table 2. The response variables, PET biodepolymerization (% weight loss) and TPA production (mM), were quantified using gravimetric analysis and HPLC, respectively. To minimize the analytical interference from other medium components, HPLC was used for accurate quantification of TPA released during optimization, as it provides higher specificity, sensitivity and selectivity compared to UV-Vis spectrophotometer^39^. Variables with significance levels above 95% were considered to enhance PET biodepolymerization.

**Table 1:** List of variables evaluated using customized screening design.

Variable code	Variable	Units	Variables evaluated for PET biodepolymerization				
X_1_	Carbon sources	1% w/v	L1 (Glucose)	L2 (Sucrose)	L3 (Lactose)	L4 (Fructose)	L5 (Xylose)
X_2_	Nitrogen sources	1% w/v	L1 (Yeast extract)	L2 (Peptone)	L3 (Casein)	L4 (Soyabean meal)	
X_3_	Surfactants	0.1% v/v	L1 (Tween 20)	L2 (Triton X-100)	L3 (SDS)		
X_4_	Basal Medium	mL	L1 (MSM)	L2 (BHB)			
X_5_	Amino acids	0.1% w/v	L1 (None)	L2 (Leucine)	L3 (Glutamine)	L4 (Cysteine)	
X_6_	PET particle size	µm	L1 (45)	L2 (90)	L3 (150)		
X_7_	PET percentage	w/v	−1 (1)	1 (5)			
X_8_	Inoculum size	v/v	−1 (5)	1 (25)			
X_9_	Agitation speed	rpm	−1 (120)	1(200)			
X_10_	pH	pH units	−1 (5)	1 (7)			

**Table 2:** An experimental design matrix with variables and responses for PET biodepolymerization by bacterial isolate *G.mysorens* ASR14.

Trial No.	Variables										PET biodepolymerization (%)		TPA produced (mM)
	X_1_	X_2_	X_3_	X_4_	X_5_	X_6_	X_7_	X_8_	X_9_	X_10_	Experimental	Predicted	
1	L1 (Glucose)	L4 (Soyabean meal)	L3 (SDS)	L2 (BHB)	L3 (Glutamine)	L2 (90)	1 (5)	−1 (5)	1 (200)	1 (7)	14.04	13.12	20.14
3	L2 (Sucrose)	L2 (Peptone)	L2 (Triton X-100)	L2 (BHB)	L1 (None)	L2 (90)	−1 (1)	−1 (5)	1 (200)	1 (7)	24.90	21.94	11.36
17	L3 (Lactose)	L2 (Peptone)	L3 (SDS)	L1 (MSM)	L2 (Leucine)	L3 (150)	1 (5)	1 (25)	1 (200)	1 (7)	31.70	31.40	42.14
15	L4 (Fructose)	L1 (Yeast extract)	L3 (SDS)	L2 (BHB)	L1 (None)	L1 (45)	1 (5)	−1 (5)	−1 (120)	1 (7)	22.40	21.81	16.02
4	L5 (Xylose)	L2 (Peptone)	L1 (Tween 20)	L2 (BHB)	L3 (Glutamine)	L1 (45)	1(5)	1 (25)	−1 (120)	−1 (5)	18.31	21.52	27.99
9	L1 (Glucose)	L2 (Peptone)	L3 (SDS)	L1 (MSM)	L4 (Cysteine)	L2 (90)	−1 (1)	−1 (5)	−1 (120)	−1 (5)	26.42	26.44	14.53
14	L2 (Sucrose)	L4 (Soyabean meal)	L1 (Tween 20)	L1 (MSM)	L4 (Cysteine)	L3 (150)	1(5)	−1 (5)	−1 (120)	1 (7)	19.50	20.92	37.57
16	L3 (Lactose)	L1 (Yeast extract)	L2 (Triton X-100)	L2 (BHB)	L3 (Glutamine)	L3 (150)	1(5)	−1 (5)	−1 (120)	−1 (5)	20.81	18.52	20.25
18	L4 (Fructose)	L3 (Casein)	L2 (Triton X-100)	L1 (MSM)	L3 (Glutamine)	L2 (90)	−1 (1)	1 (25)	−1 (120)	1 (7)	13.80	9.18	12.07
11	L5 (Xylose)	L4 (Soyabean meal)	L2 (Triton X-100)	L1 (MSM)	L2 (Leucine)	L1 (45)	−1 (1)	−1 (5)	−1 (120)	1 (7)	6.70	2.90	14.74
13	L1 (Glucose)	L3 (Casein)	L2 (Triton X-100)	L1 (MSM)	L1 (None)	L1 (45)	1(5)	1 (25)	1 (200)	−1 (5)	31.41	26.83	35.67
10	L2 (Sucrose)	L3 (Casein)	L3 (SDS)	L2 (BHB)	L2 (Leucine)	L2 (90)	1(5)	1 (25)	−1 (120)	−1 (5)	2.10	7.02	7.17
2	L3 (Lactose)	L4 (Soyabean meal)	L1 (Tween 20)	L1 (MSM)	L1 (None)	L2 (90)	−1 (1)	1 (25)	−1 (120)	−1 (5)	7.00	9.51	2.15
7	L4 (Fructose)	L1 (Yeast extract)	L1 (Tween 20)	L1 (MSM)	L2 (Leucine)	L2 (90)	1(5)	−1 (5)	1 (200)	−1 (5)	28.41	28.40	38.88
12	L5 (Xylose)	L3 (Casein)	L3 (SDS)	L1 (MSM)	L1 (None)	L3 (150)	−1 (1)	−1 (5)	1 (200)	−1 (5)	12.62	18.21	15.34
8	L1 (Glucose)	L1 (Yeast extract)	L1 (Tween 20)	L2 (BHB)	L2 (Leucine)	L3 (150)	−1 (1)	1 (25)	−1 (120)	1 (7)	7.40	6.58	12.81
19	L2 (Sucrose)	L1 (Yeast extract)	L3 (SDS)	L1 (MSM)	L3 (Glutamine)	L1 (45)	−1 (1)	1 (25)	1 (200)	−1 (5)	18.50	23.09	9.46
20	L3 (Lactose)	L3 (Casein)	L1 (Tween 20)	L2 (BHB)	L4 (Cysteine)	L1 (45)	−1 (1)	−1 (5)	1 (200)	1 (7)	14.92	13.55	13.98
6	L4 (Fructose)	L4 (Soyabean meal)	L2 (Triton X-100)	L2 (BHB)	L4 (Cysteine)	L3 (150)	−1 (1)	1 (25)	1 (200)	−1 (5)	9.80	10.59	12.05
5	L5 (Xylose)	L1 (Yeast extract)	L2 (Triton X-100)	L1 (MSM)	L4 (Cysteine)	L2 (90)	1(5)	1 (25)	1 (200)	1 (7)	38.70	37.80	43.69

X_1_: Carbon sources, 1% w/v; X_2_: Nitrogen sources, 1% w/v; X_3_: Surfactants, 0.1% v/v; X_4_: Basal Medium; X_5_: Amino acids, 0.1% w/v; X_6_: PET powder size, µm; X_7_: PET percentage, w/v; X_8_: Inoculum size, v/v; X_9_: Agitation speed, rpm; X_10_: pH, pH units.

### Central composite design to optimize the key variables

The significant variables identified through the customized screening design: PET powder size, nitrogen source (peptone), PET concentration and incubation time were further optimized using CCD. A four-variable with a five-level (−2, −1, 0, + 1, +2) of 25 trials was employed to correlate significant variables and responses to find the optimal point for enhanced PET biodepolymerization. The maximum and minimum levels of the variables used in CCD are shown in Table 3. The experimental design matrix with actual and coded values is presented in Table 4. To predict the optimal conditions, a quadratic Eq. (1) was fitted to correlate the relationship between the independent variables and the response.

1 $$\begin{aligned} {\mathrm{Y}} = & {\text{ }}\beta _{0} + \beta _{{\mathrm{1}}} {\mathrm{Y}}_{{\mathrm{1}}} + {\text{ }}\beta _{{\mathrm{2}}} {\mathrm{Y}}_{{\mathrm{2}}} + {\text{ }}\beta _{{\mathrm{3}}} {\mathrm{Y}}_{{\mathrm{3}}} + {\text{ }}\beta _{{\mathrm{4}}} {\mathrm{Y}}_{{\mathrm{4}}} + {\text{ }}\beta _{{{\mathrm{11}}}} {\mathrm{Y}}_{{\mathrm{1}}} ^{{\mathrm{2}}} \\ & + {\text{ }}\beta _{{{\mathrm{22}}}} {\mathrm{Y}}_{{\mathrm{2}}} ^{{\mathrm{2}}} + {\text{ }}\beta _{{{\mathrm{33}}}} {\mathrm{Y}}_{{\mathrm{3}}} ^{{\mathrm{2}}} + {\text{ }}\beta _{{{\mathrm{44}}}} {\mathrm{Y}}_{{\mathrm{4}}} ^{{{\mathrm{2}}~~}} \beta _{{{\mathrm{12}}}} {\mathrm{Y}}_{{\mathrm{1}}} {\mathrm{Y}}_{{\mathrm{2}}} + {\text{ }}\beta _{{{\mathrm{13}}}} {\mathrm{Y}}_{{\mathrm{1}}} {\mathrm{Y}}_{{\mathrm{3}}} \\ & + {\text{ }}\beta _{{{\mathrm{14}}}} {\mathrm{Y}}_{{\mathrm{1}}} {\mathrm{Y}}_{{\mathrm{4}}} + {\text{ }}\beta _{{{\mathrm{23}}}} {\mathrm{Y}}_{{\mathrm{2}}} {\mathrm{Y}}_{{\mathrm{3}}} + {\text{ }}\beta _{{{\mathrm{24}}}} {\mathrm{Y}}_{{\mathrm{2}}} {\mathrm{Y}}_{{\mathrm{4}}} + {\text{ }}\beta _{{{\mathrm{34}}}} {\mathrm{Y}}_{{\mathrm{3}}} {\mathrm{Y}}_{{\mathrm{4}}} ^{{}} \\ \end{aligned}$$

Where Y is the response (% weight loss), β_0_ is the constant, Y_1_ (peptone, w/v), Y_2_ (PET Concentration, w/v), Y_3_ (time, d), and Y_4_ (PET particle size, µm) are independent variables, β_1_, β_2_, β_3,_ and β_4_ are linear coefficients, β_12_, β_13_, β_14_, β_23_, β_24_, and β_34_ are interaction coefficients, and β_11_, β_22_, β_33_, and β_44_ are quadratic coefficients.

**Table 3:** Variables and their levels used in CCD for PET biodepolymerization with bacterial isolate *G. mysorens* ASR 14.

Variable code	Variables	Units	Low		Centre	High	
			−2	−1	0	+ 1	+ 2
Y_1_	Peptone	w/v	0	0.5	1	1.5	2
Y_2_	PET concentration	w/v	1	2	3	4	5
Y_3_	Time	d	20	30	40	50	60
Y_4_	PET particle size	µm	45	90	150	300	

**Table 4:** CCD with experimental and predicted data.

Trial No.	Variables				PET biodepolymerization (%)		TPA produced (mM)
	Y_1_	Y_2_	Y_3_	Y_4_	Experimental	Predicted	
1	0.5	2	40	150	31.80	29.06	23.94
2	1.5	3	50	150	34.39	30.98	32.43
3	2	5	50	45	72.80	69.36	41.90
4	2	1	60	150	25.60	24.63	2.58
5	0	5	50	150	26.17	29.65	36.31
6	0.5	2	30	150	31.61	28.83	20.52
7	0	1	20	90	38.54	39.18	12.36
8	0.5	4	50	300	31.41	25.85	33.91
9	0	1	60	300	37.07	36.36	12.41
10	2	1	20	300	11.74	6.92	11.03
11	0	3	40	45	40.70	41.66	29.62
12	0.5	1	60	45	37.82	37.07	16.34
13	1	1	40	300	17.01	24.77	10.80
14	1	4	60	90	34.73	39.90	22.15
15	1.5	2	20	45	32.71	34.04	21.30
16	2	1	40	45	32.20	34.18	13.14
17	1.5	5	30	300	26.80	27.48	35.13
18	0.5	5	20	45	50.91	48.06	36.44
19	0	3	20	300	30.94	32.35	23.29
20	2	4	20	150	29.10	31.60	31.47
21	2	3	60	300	23.83	26.04	29.38
22	1.5	4	30	150	29.54	32.72	32.86
23	1	5	60	300	28.10	27.50	37.42
24	1	2	50	150	29.61	27.08	18.77
25	0.5	4	30	150	31.20	30.93	32.64

Y_1_: Peptone, w/v; Y_2_: PET concentration, w/v; Y_3_: Time, d; Y_4_: PET particle size, µm.

### Statistical analysis

The statistical fitness of the model was determined by Analysis of Variance (ANOVA). The efficiency of the model was checked using the sum of squares, degrees of freedom, p-value, and F-value. The regression coefficient was evaluated to determine the extent of the fit. Response surface and variable interaction plots were generated to visualize the simultaneous effect of the variables on the response.

### PET biodepolymerization studies using crude enzyme extract

PET powder with a particle size of 45 μm was sterilized with 90% (v/v) ethanol and used as the substrate. The pre-weighed PET powder (100 mg) was added to an Erlenmeyer flask containing 10 mL of crude enzyme and incubated at 32 °C and 200 rpm for 10 d. The biodepolymerization potential of the crude enzyme was evaluated by the weight loss method and HPLC to quantify TPA.

### Analytical methods

The residual PET powder and TPA in the cell-free extracts of the highest PET biodepolymerization trials from the customized screening design (Trial-5) and CCD (Trial-3) were analyzed using spectrophotometry and chromatography to assess the progressive depolymerization of PET. The residual PET powder was characterized by SEM, FTIR, Raman spectroscopy, and XRD, whereas the TPA released was analyzed using UV-Vis spectroscopy, TLC, HPLC, NMR, and GC-MS. Commercial PET powder and TPA were used as the controls.

#### Determination of PET biodepolymerization by gravimetric weight loss method

The culture medium was centrifuged at 10,000 rpm for 10 min to separate the residual PET powder. The supernatant was preserved at 4 °C to quantify TPA. Toluene was added to the pellet to disintegrate the biomass, centrifuged at 10,000 rpm for 15 min and filtered using Whatman qualitative filter paper, Grade 1 (11 μm). The filter paper was weighed before and after filtration (Sartorius Analytical Balance- Quintix 224). The residue was dried in a hot air oven at 50 °C to determine the weight of the residual PET powder^40^. The extent of biodepolymerization was calculated using Eq. (2)

2 $$\:\mathrm{PET\:percentage\:weight\:loss=}\frac{\mathrm{Initial\:weight-Final\:weight}}{\mathrm{Initial\:weight}}\mathrm{\:\:X\:100}$$

#### Scanning electron microscopy analysis

The residual PET powder was collected to assess the morphology before and after biodepolymerization. The samples were sputtered with gold, and scanning electron microscopy was performed using a Zeiss SEM EV018 at an accelerating voltage of 10 kV. The elemental compositions were compared using EDS. All elements were analyzed using four iterations.

#### Thin layer chromatography

TLC was used to validate the release of TPA upon biodepolymerization of PET powder. It was performed on aluminum sheets of silica gel 60 F_254_ (Merck) as a stationary phase. The mobile phase comprised methanol, acetonitrile, and water in a ratio of 2:3:5. A standard solution of 1 mg/mL commercial TPA in MSM was used as a positive control, and uninoculated MSM was used as a negative control. Cell-free supernatants of Trial-5 (43.80%) of customized screening and Trial-3 (75.6%) of CCD were used as the test samples. The TLC plates were air-dried, and TPA spots were detected using a UV lamp at 254 nm.

#### Fourier transform infrared spectroscopy

FTIR was used to evaluate the shifts in spectral peaks and changes in the functional groups of the residual PET powder by the bacterial isolate *G. mysorens* ASR 14. FTIR analysis was performed using a Shimadzu spectrometer 00254 via ATR probing over a 470–4000 cm^–1^ wavelength range at 4 cm^–1^ resolution.

#### High-performance liquid chromatography

The cell-free extract was diluted 20 times with deionized water and filtered using a 0.22 μm syringe filter. For TPA quantification, 20 µL of the filtered solution was manually injected into the HPLC system (RP-HPLC Shimadzu LC-20AD). A reverse-phase C18 column was used to separate the sample mixture. Acetonitrile and 0.1% formic acid in a 40:60 v/v binary gradient were used as the mobile phase. The column temperature and flow rate were maintained at 25 °C and 1 mL/min, respectively, at 240 nm, following slight modifications as described by Puspitasari et al.^41^ The analysis was performed in triplicates. The TPA standards in MSM were prepared at a concentration of 500 µg/mL and serially diluted for calibration (Fig. S3). The limit of detection (LOD) and limit of quantification (LOQ) were calculated based on the standard deviation of triplicates and the slope of the calibration curve.

#### Gas chromatography-mass spectroscopy

GC-MS analysis of the cell-free extracts of the untreated and treated PET samples was performed to measure the profiles of volatile and semi-volatile organic compounds. GC-MS was performed using an Agilent system equipped with a PAL 3 autosampler and a DB-5ms capillary column. The injection volume was 1 µL in splitless mode using a 10 µL syringe (Agilent 8010 − 1308) with an inlet temperature set to 250 °C. The column temperature was maintained at 350 °C, with helium as the carrier gas at a flow rate of 0.7783 mL/min. Mass spectra were captured using electron ionization mode, and the analytes were identified by a mass-selective detector. Data acquisition was performed over a full scan range with a chromatographic run time of 20 min.

#### Raman spectroscopy

Raman Spectroscopy was used to analyze the extent of chemical changes in the polymer and identify the depolymerization products. The instrument used was the B&W Tek i-Raman^®^ Plus Portable Fiber Optic Raman System, equipped with a 785 nm excitation laser and a maximum power output of 350 mW. Three spectra were recorded for each sample and processed using OriginPro 2024 software. The spectra were averaged and baseline-corrected using Asymmetric Least Squares (ALS) smoothing and subsequently smoothed to obtain fully processed Raman spectra.

#### Nuclear magnetic resonance spectroscopy

Proton (^1^H) and carbon (^13^C) NMR were performed to analyze the end products. Cell-free extracts from Trial-5 (Table 2) and Trial-3 (Table 4) were used as test samples, and uninoculated MSM was used as the control. The analysis was performed using a Burker Spectrometer. The cell-free extract was dissolved in DMSO-d6 and filtered through a 0.22 μm PES syringe filter. The samples were transferred to NMR tubes for analysis. The chemical shifts were compared with the control spectra.

#### X-ray diffraction

The crystallinity of PET powder before and after biodepolymerization was determined using a Rigaku Ultima IV diffractometer with a Cu Kα radiation source with a wavelength of 1.540562 Å. The PET powder samples were analyzed in an angle range of 2θ from 5° to 90° with a scan rate of 2° per minute. The diffractograms were analyzed to determine the peak positions and crystallinity. The crystallinity index was calculated using Eq. 3.

3 $$\:\mathrm{CI\:(\%)=}\frac{\mathrm{I}\mathrm{max}\mathrm{-I}\mathrm{min}}{\mathrm{I}\mathrm{max}}\mathrm{\:X\:100\:}$$

I_max_ = the maximum peak intensity, and I_min_= height of the minima observed above the baseline.

## Results and discussion

### Screening, characterization, and identification of the PET depolymerizing bacterial isolate

**Fig. 1:**
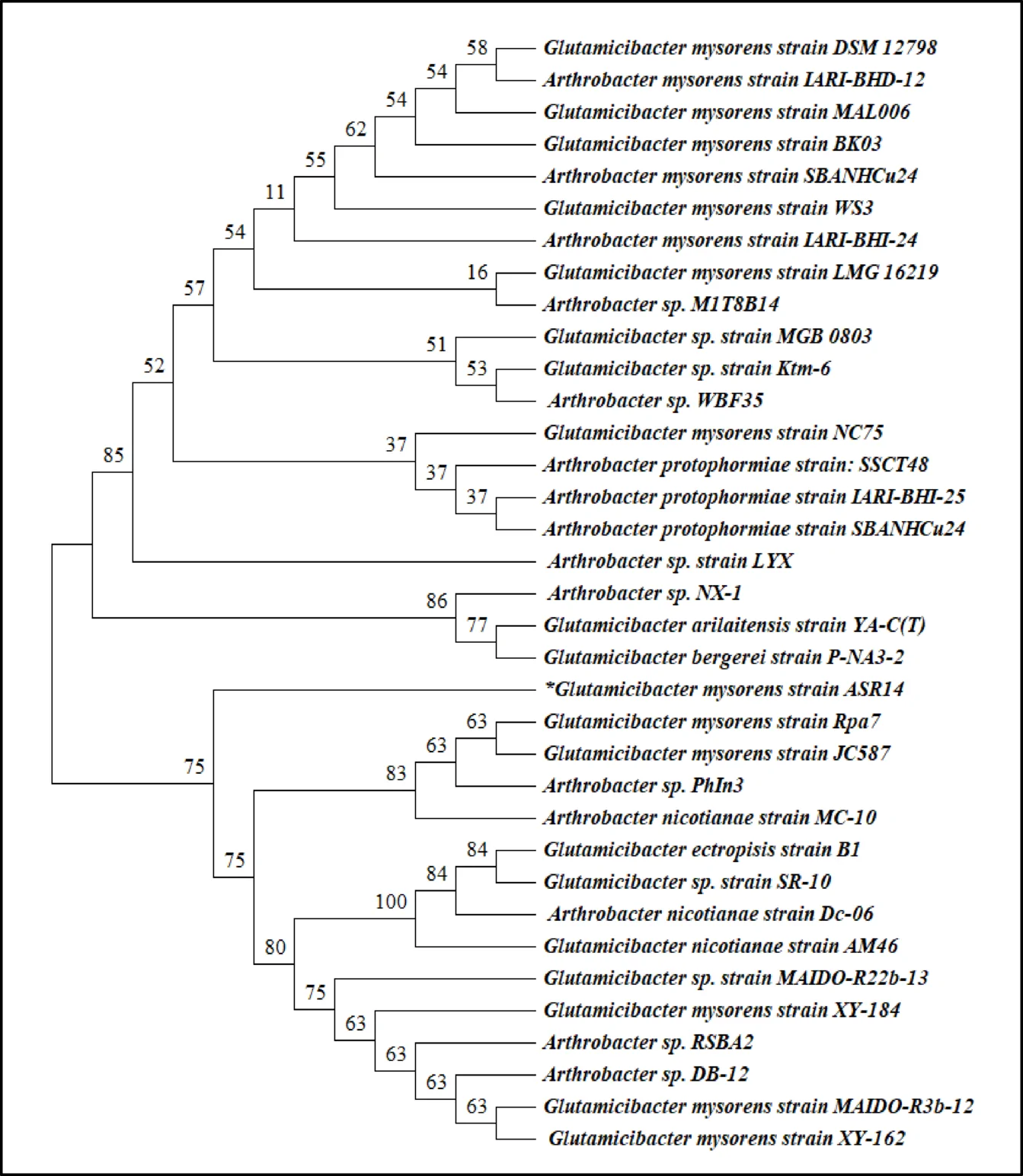
Identification of the PET-degrading bacterial isolate 14 as *G. mysorens* ASR14 based on the neighbour-joining method.

**Fig. 2:**
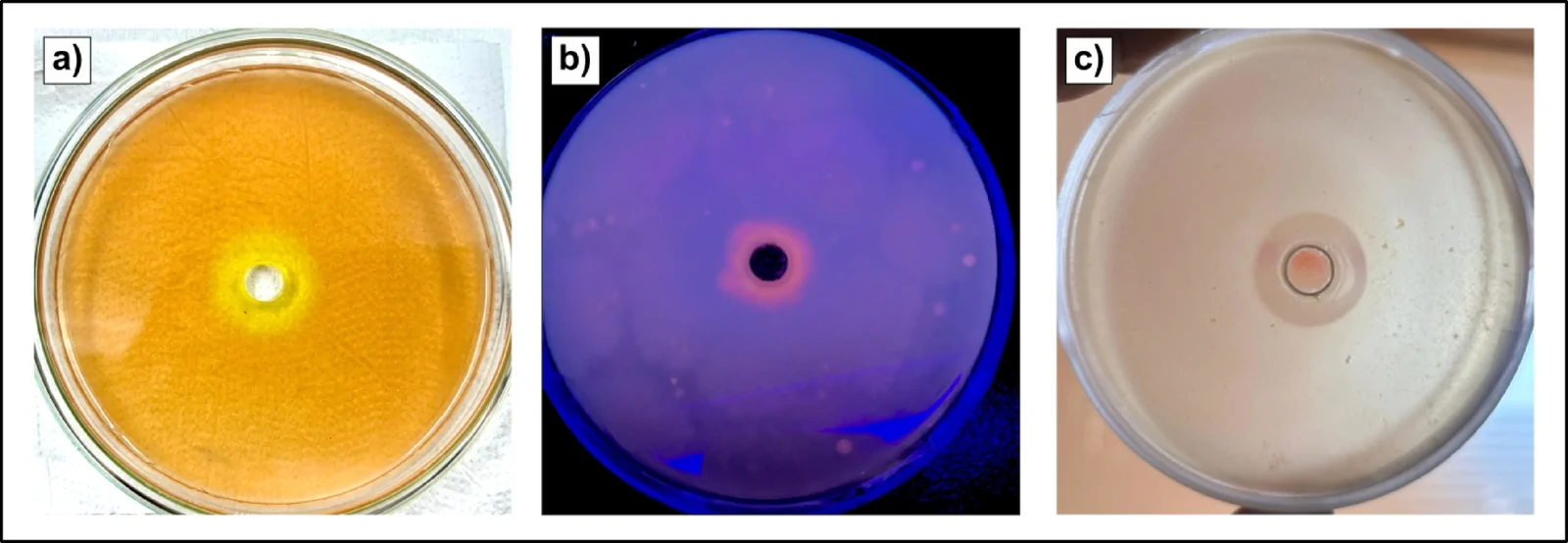
Biochemical characterization of *G. mysorens* ASR 14 enzymes for PET biodepolymerization: (**a**) Esterase plate assay (**b**) Lipase assay (**c**) Protease assay.

**Fig. 3:**
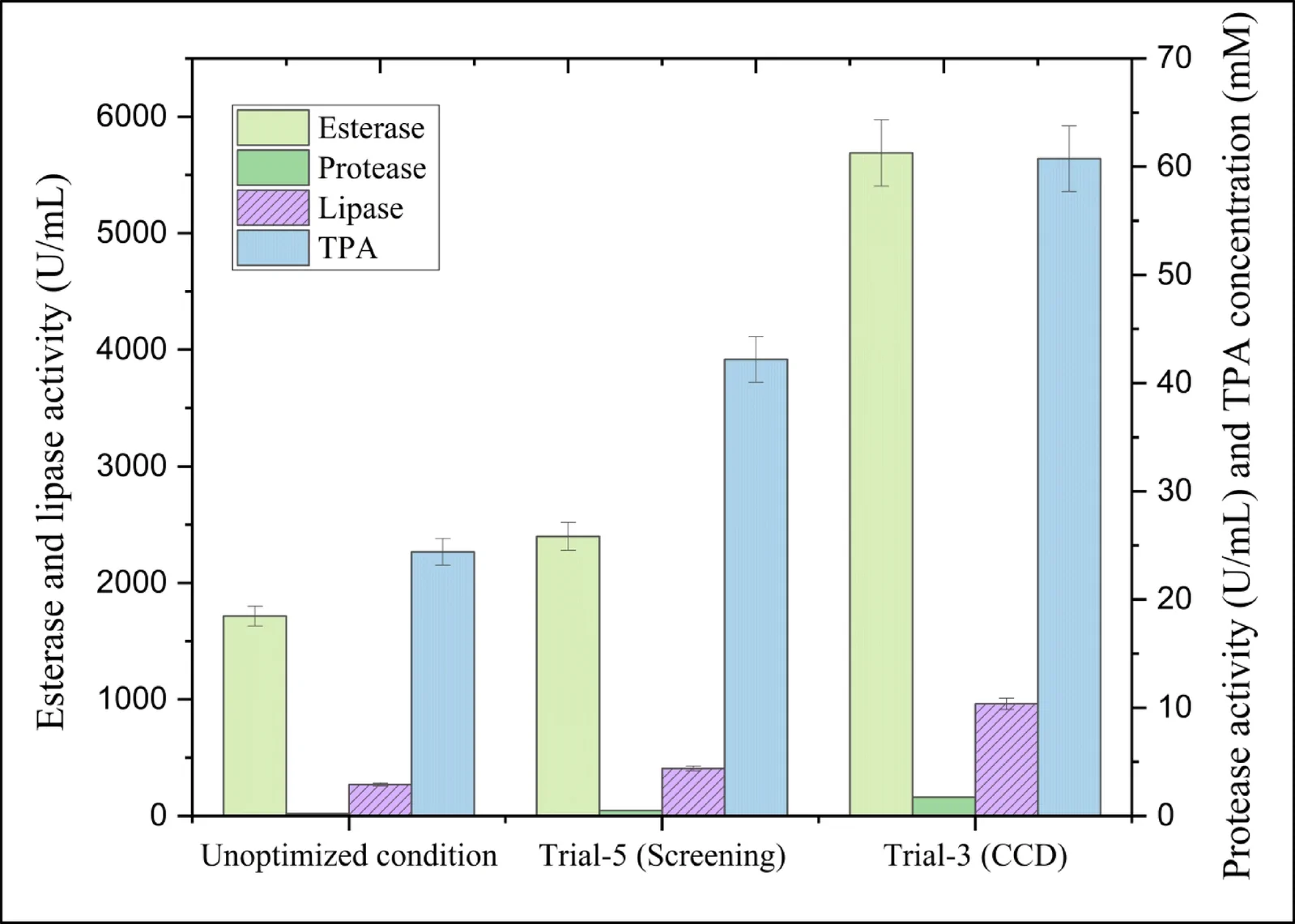
Quantification of enzyme activities involved in all stages of statistical optimization of commercial PET powder biodepolymerization.

PET-degrading bacteria were isolated using the liquid enrichment method, followed by spread plating on a 1% PET-MSM agar plate^42,43^. Fifteen morphologically distinct bacterial isolates were screened for PET depolymerization in 1% PET-MSM liquid media based on weight loss analysis and quantification of TPA release. Among all the isolates, isolate 14 showed 27.6% PET depolymerization and a maximum TPA concentration of 24.40 mM in 30 d. Isolate 14 exhibited a Gram-positive and rod shape during the log phase and a coccoid shape in the mature phase^44^. It was identified based on the 16 S rRNA gene nucleotide sequence, which showed 98.74% Similarity with *Glutamicibacter mysorens*. The 1200 bp gene sequence was submitted to GenBank under accession number PQ813653 (Fig. S4). A phylogenetic tree was constructed using MEGA 11 software, as shown in Fig. 1. Biochemical assays were performed for preliminary phenotypic identification and physiological characterization of *G.mysorens* ASR 14. The isolate was positive for catalase, methyl red, indicating oxidative stress tolerance and the ability to use mixed-acid fermentation pathways, reflecting metabolic adaptability under diverse environmental conditions. Negative tests for VP, IAA, TSI, and starch hydrolysis of the bacterium exhibited a lack of butanediol fermentation, sulfide production and extracellular amylase activity with non-fermentative metabolism, indicating the utilization of other unconventional carbon sources (Fig. S5)^45^. The enzymatic activity of *G. mysorens* ASR14 was qualitatively analyzed using agar plate assays for esterase, lipase, and protease. The esterase assay was evident from a yellow halo within 15 min, indicating the release of p-nitrophenol and acetic acid^32^. Lipase activity was confirmed by the formation of an orange fluorescent halo under UV light due to the interaction of free fatty acids with Rhodamine B^46^. Protease activity was observed as a clear zone of hydrolysis (Fig. 2). The enzymatic activity was analyzed under unoptimized conditions, initial screening (Trial-5), and CCD (Trial-3) (Fig. 3). Esterase activity increased from 1715 U/mL to over 5690 U/mL in the CCD-optimized trial. Lipase activity also increased across the trials to 962 U/mL, whereas the protease activity remained low throughout optimization. The TPA concentration, which is indicative of PET hydrolysis, increased from 24.40 mM to 60.74 mM. The trends demonstrated that process optimization enhanced both the enzyme activity profile and PET biodepolymerization. The esterase, lipase and protease activities exhibited by *G.mysorens* ASR 14 suggest the role of extracellular enzymes in PET depolymerization. However, the specific enzymes (PETase and MHETase) involved were not identified in the present study and require further validation through genomics and biochemical characterization.

### Screening of media components for an optimized PET biodepolymerization

In the experimental customized screening of variables, PET biodepolymerization ranged from 2.9% to 37.8%, reflecting the role of basal media composition and other process variables in enhancing the efficiency (Table 2). Trial-5 exhibited a maximum PET weight loss of 38.7% in 20 days. The R^2^ value for the screening of variables was 0.90, with a p-value of 0.0005 (Fig. S6), implying that the selected variables could explain 90% of the model. The design showed that the variables X_7_: PET percentage, (X_2_ L2): nitrogen source-peptone, (X_4_ L1): culture medium, X_9_: Agitation speed, and (X_5_ L4): amino acid (cysteine) had a statistically positive effect on the response. The parameter estimates from the customized screening design demonstrated that medium composition and process variables influence PET biodepolymerization. Positive coefficients indicated factors that enhanced the biodepolymerization, whereas negative coefficients showed inhibitory effects on the response. The highly significant variables (at 95% significance) with their corresponding p-values are presented in Table S1. The carbon sources had an insignificant effect on PET biodepolymerization, indicating the potential of *G. mysorens* ASR 14 to utilize PET-derived substrates as the carbon source^47^. *Glutamicibacter* strains PB8-1 and BO25 exhibited growth in MSM supplemented with TPA, a PET monomer^48^. Additionally, studies have shown that bacteria can degrade PET without additional carbon sources^49^. Among the nitrogen sources evaluated, peptone exhibited the highest positive coefficient (+ 7.58, *p* = 0.0066) followed by yeast extract (+ 3.94, *p* = 0.0397). Peptone provides readily assimilable peptides, amino acids, vitamins and micronutrients that support microbial growth and improve nutrient availability, balancing the carbon-to-nitrogen ratio, enhancing microbial activity and allowing better PET biodepolymerization^50,51^. Soybean meal and casein negatively affected the response due to the particulate nature and slower nutrient accessibility, reducing nutrient uptake and interfering with bacterial metabolism. Triton X-100, a non-ionic surfactant, showed a positive impact (+ 2.70, *p* = 0.054) by enhancing polymer surface wettability, reducing surface tension, and increasing microbial access to hydrophobic substrate by improving PET-water interactions^25,52^. This aligns with the findings of Fu et al., who reported that surfactants can increase the adsorption of enzymes on the PET substrate, thereby increasing the depolymerization rate^29^. Tween 20 and SDS exhibited a negative coefficient on PET biodepolymerization which might be due to their anionic detergent properties, which can disrupt membrane integrity and affect bacterial viability and enzyme activity^53^. The essential inorganic nutrients in MSM promoted cellular metabolism and enzyme production. PET concentration at higher loading provided greater surface area and improved enzyme activity^54^. The agitation improved oxygen transfer, nutrient distribution and contact between the substrate and enzyme^55^. The other variables, inoculum, pH and incubation time had negligible effects on the response. The model was validated by employing the optimized variable values with and without the carbon source, as determined by the software for 30 d (Table 5). The validated experiment without a carbon source showed higher PET biodepolymerization (43.8%). This suggested that when two carbon sources are available simultaneously, microbes prefer simpler carbon sources that require less energy than complex polymers that require more energy for utilization^56,57^.

**Table 5:** Experimental validation of variables on PET biodepolymerization.

Setting	Variables										PET biodepolymerization (%)		TPA produced (mM)
	X_1_	X_2_	X_3_	X_4_	X_5_	X_6_	X_7_	X_8_	X_9_	X_10_	Experimental	Predicted	
Optimal with carbon source	L1	L2	L2	L1	L4	L1	1	−1	1	1	39.20 ± 0.21	49.39	40.11
Optimal without carbon source	-	L2	L2	L1	L4	L1	1	−1	1	1	43.80 ± 0.30	45.39	42.16

X_1_(L1): Glucose, 1% w/v; X_2_(L2): Peptone, 1% w/v; X_3_(L2): Triton X-100, 0.1% v/v; X_4_ (L1):MSM; X_5_ (L4): Cysteine, 0.1% w/v; X_6_ (L1):PET powder size, 45 μm; X_7_:PET percentage, 5 g; X_8_:Inoculum size, 5% v/v; X_9_:Agitation speed, 200 rpm; X_10_:pH, 7.

### Customized central composite design for optimizing key variables

In the next optimization step, a four-variable, five-level customized CCD with 25 trials was employed using the significant variables from the screening testing: nitrogen source, PET percentage, PET particle size, and time, with other significant variables kept constant (Table 3). The measured responses were PET weight loss (%) and TPA production (mM). Table 4 shows that Trial-3 exhibited the highest PET depolymerization (72.8%) in 50 d, whereas the lowest depolymerization was observed in Trial-10 (11.74%). The variability in PET weight loss in different trials highlights the importance of studying the effects of nutritional and physical variables. Trials with PET powder of 45 μm particle size showed maximum depolymerization compared to PET powder with a larger particle size. Peptone improves microbial activity in basal media by providing free peptides^58^. Lower PET concentrations and smaller particle sizes resulted in better depolymerization^59^. Initially, nutrient abundance accelerated the process; however, the accumulation of reaction by-products over time hindered biodepolymerization efficiency^60^. The statistical analysis of this data determined the R^2^ value to be 0.92, with a p-value of 0.0009 (Fig. S7). The residual analysis for the developed model is presented in Fig S8. A second-order polynomial function was fitted to the experimental design matrix, and the regression equation is shown in Eq. 4.

4 $$\begin{aligned} {\mathrm{Z}} = & {\text{ 27}}.0{\mathrm{6}} - 0.{\mathrm{72Y}}_{{\mathrm{1}}} + {\mathrm{5}}.0{\mathrm{9Y}}_{{\mathrm{2}}} + {\mathrm{1}}.{\mathrm{63}}0{\mathrm{Y}}_{{\mathrm{3}}} - {\mathrm{9}}.{\mathrm{29Y}}_{{\mathrm{4}}} + {\mathrm{2}}.{\mathrm{43Y}}_{{\mathrm{1}}} ^{{\mathrm{2}}} + {\mathrm{2}}.0{\mathrm{6Y}}_{{\mathrm{2}}} ^{{\mathrm{2}}} \\ & - {\mathrm{1}}.{\mathrm{44Y}}_{{\mathrm{3}}} ^{{\mathrm{2}}} + {\mathrm{6}}.{\mathrm{33Y}}_{{\mathrm{4}}} ^{{\mathrm{2}}} + {\text{ 7}}.{\mathrm{68Y}}_{{\mathrm{1}}} {\mathrm{Y}}_{{\mathrm{2}}} - {\text{ 4}}.{\mathrm{18Y}}_{{\mathrm{1}}} {\mathrm{Y}}_{{\mathrm{4}}} \\ & + {\text{ 4}}.{\mathrm{11Y}}_{{\mathrm{1}}} {\mathrm{Y}}_{{\mathrm{3}}} - {\text{ 3}}.{\mathrm{69Y}}_{{\mathrm{2}}} {\mathrm{Y}}_{{\mathrm{4}}} - {\text{ }}0.{\mathrm{38Y}}_{{\mathrm{2}}} {\mathrm{Y}}_{{\mathrm{3}}} + {\text{ }}0.{\mathrm{12Y}}_{{\mathrm{3}}} {\mathrm{Y}}_{{\mathrm{4}}} \\ \end{aligned}$$

Where Z represents the percentage weight loss of PET powder, Y_1_, Y_2_, Y_3_, and Y_4_ are the coded values for peptone (w/v), PET concentration (w/v), time (d), and PET particle size (µm), respectively. Model fitness was determined using ANOVA, as shown in Table 6. The model was statistically significant, with an F-value of 8.36 and a corresponding p-value of 0.0009. Among the individual variables, PET concentration (Y_2_) and PET particle size (Y_4_) were the most crucial variables, with F values of 14.18 (*p* = 0.0037) and 48.80 (*p* < 0.0001), respectively. The quadratic term Y_4_^**2**^ was also significant (*p* = 0.0241). The two-factor interaction between peptone and PET concentration (Y_1_Y_2_) had the highest interaction F-value of 18.81 (*p* = 0.0015), which was clearly visualized in the saddle-shaped surface plot (Fig. 5). Similarly, the interactions of peptone and time (Y_1_Y_3_; *p* = 0.0393), peptone and PET particle size (Y_1_Y_4_; *p* = 0.034), and PET concentration and time (Y_2_Y_3_; *p* = 0.0434) were also significant. The interactions between PET particle size and time (Y_4_ Y_3_) and PET concentration and time (Y_2_Y_3_) were statistically insignificant, highlighting the minimal influence.

**Table 6:** ANOVA for central composite data design for PET biodepolymerization.

Source	DF	Sum of squares	F-value	*p*- value
Model	14	2805.0143	8.3616	0.0009*
Y_1_	1	6.5615	0.2738	0.6122
Y_2_	1	339.8753	14.184	0.0037*
Y_3_	1	34.6184	1.4447	0.2571
Y_4_	1	1169.395	48.8024	< 0.0001*
Y_1_^2^	1	21.2958	0.8887	0.368
Y_2_^2^	1	15.5931	0.6507	0.4386
Y_3_^2^	1	7.8568	0.3279	0.5796
Y_4_^2^	1	168.9941	7.0526	0.0241*
Y_1_*Y_2_	1	450.6808	18.8083	0.0015*
Y_1_*Y_3_	1	134.4725	5.6119	0.0393*
Y_1_*Y_4_	1	144.3833	6.0256	0.034*
Y_2_*Y_3_	1	1.1564	0.0483	0.8305
Y_2_*Y_4_	1	127.9554	5.34	0.0434*
Y_4_*Y_3_	1	0.1386	0.0058	0.9409
Error	10	239.6183		
C. Total	24	3044.6327		

**Fig. 4:**
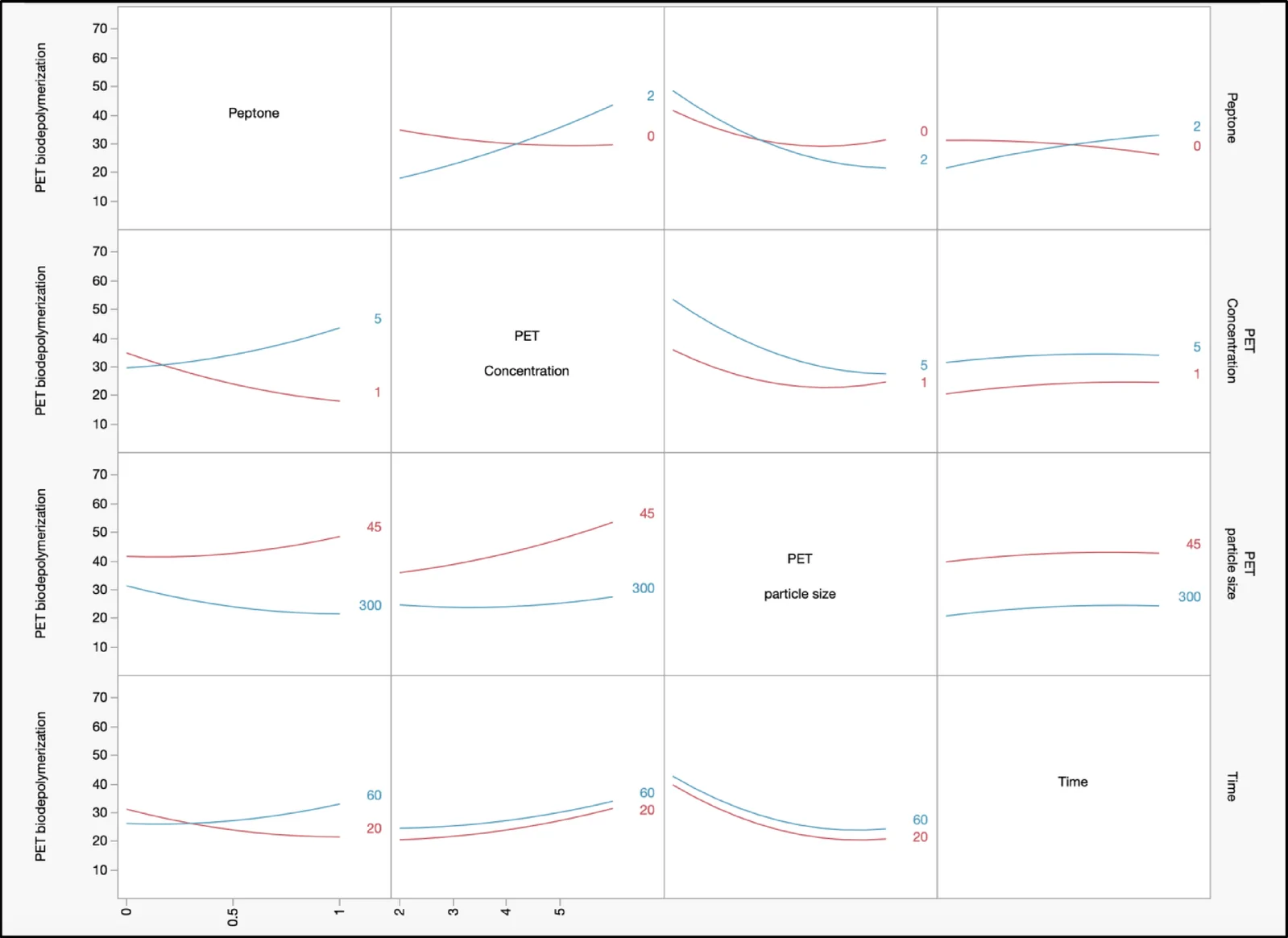
Interaction plots illustrating the effects of peptone, PET concentration, PET particle size, and incubation time on PET biodepolymerization (%).

**Fig. 5:**
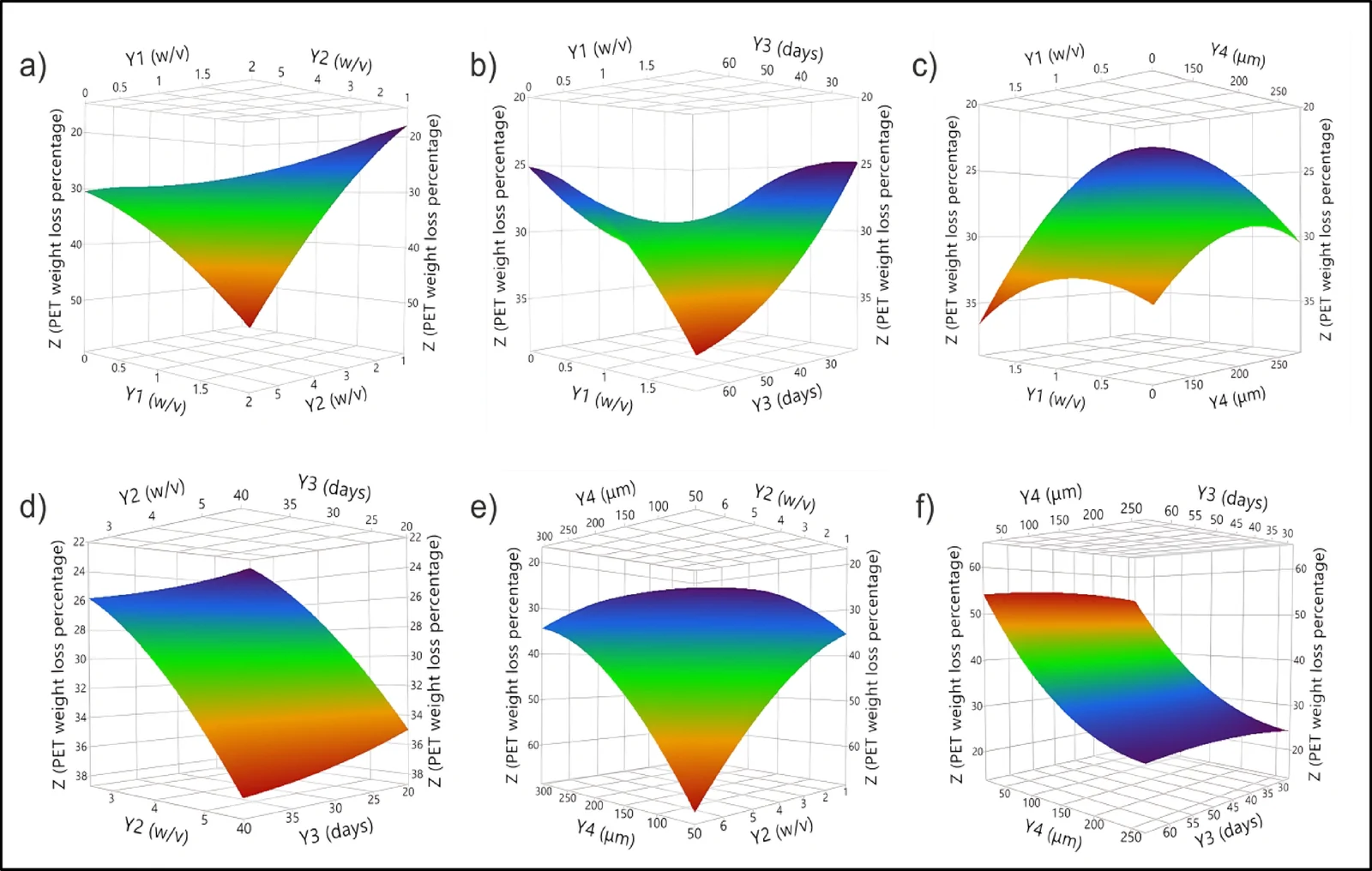
Surface plots depicting the interaction effects of different variables on PET biodepolymerization (**a**) Peptone concentration (% w/v) and PET concentration (% w/v) (**b**) Peptone concentration (% w/v) and Time (d) (**c**) Peptone concentration (% w/v) and PET particle size (µm) (d) PET concentration (% w/v) and Time (d) (**e**) PET particle size (µm) and PET concentration (% w/v) (**f**) PET particle size (µm) and Time (d).

The interaction effects of four independent variables, peptone concentration, PET concentration, PET particle size, and incubation time, on the percentage of PET biodepolymerization were analyzed using interaction plots (Fig. 4), and 3D surface profiler plots (Fig. 5). The subplots showed two colour-coded lines that distinguished the responses at the coded levels. The orientation of the lines determined the interaction between two variables. Parallel lines suggested minimal or no interaction, whereas converging or intersecting lines indicated an interaction effect^61^. Interaction between peptone and PET concentration showed a synergistic influence on PET biodepolymerization. At lower PET concentrations, increasing peptone reduced biodepolymerization, whereas at higher concentrations of PET, peptone efficiently enhanced biodepolymerization. The saddle-shaped response surface plot confirmed that the maximum PET weight loss was achieved at elevated peptone concentration (Fig. 5a). A significant interaction was also observed between peptone concentration and incubation time. Prolonged incubation improved depolymerization at a higher peptone concentration. Initially, PET biodepolymerization remained relatively moderate, with increased incubation time, there was an increased secretion of enzymes which can be attributed to the adaptation of *G.mysorens* ASR 14 to PET as he carbon source (Fig. 5b). A significant interaction was observed between peptone concentration and PET particle size although the lines did not intersect (Fig. 4). The increase in peptone concentration enhanced depolymerization at smaller PET particle sizes, which was attributed to a higher surface area-to-volume ratio. The concentration and size of PET showed significant interactions (Fig. 5c). Lower concentrations and smaller particle sizes exhibited better depolymerization^62^. The interaction between PET particle size and incubation time showed parallel lines, indicating that microbial depolymerization occurred at a relatively constant rate. The interaction between PET concentration and incubation time influenced PET biodepolymerization (Fig. 5 d). At shorter incubation time, increasing the PET concentration did not improve biodepolymerization. After prolonged incubation, higher PET concentrations resulted in higher PET loss. A significant influence of PET particle size and PET concentration suggested that smaller to intermediate particle sizes with moderate PET concentration provide larger surface area, whereas at larger particle sizes and high PET concentrations, biodepolymerization decreased (Fig. 5e). The interaction between PET particle size and incubation time in Fig. 5f showed that depolymerization efficiency improved with increasing time and decreasing particle size The A 3D response surface plot was generated from the fitted quadratic model. The validation of the present study showed a 2.73-fold increase in biodepolymerization under optimal conditions: peptone (2% w/v), PET concentration (5% w/v), PET particle size 45 μm, and time (60 d). Under these conditions, 75.6% depolymerization was obtained, producing 60.74 mM of TPA, surpassing several previously reported bacterial strains for depolymerizing PET microplastics^9,26,27^. While a reduction in PET weight and TPA release was observed, the analysis mainly indicates PET depolymerization rather than total mineralization, as carbon balance analysis, Total Organic Carbon (TOC) reduction, CO_2_ evolution and comprehensive profiling of depolymerization intermediates (MHET, BHET) were not performed. Therefore, complete metabolic assimilation of PET-derived carbon cannot be confirmed under the experimental conditions employed in this study.

Previous studies have demonstrated the ability of bacterial strains to depolymerize PET under different experimental conditions. *Gordonia* sp. CN2K degraded 40.43% of PET microplastics (< 5 mm) in 45 days^63^, while *Streptomyces* sp. achieved degradation of 49.2% (500 μm), 57.48% (420 μm), 62.48% (300 μm), and 68.8% (212 μm), confirming enhanced degradability at lower particle sizes^40^. *Enterobacter hormaechei* exhibited 60% reduction in the carbon content of PET microplastics after 28 days of incubation^64^. A bacterial consortium of *Priestia aryabhattai*,* Bacillus pseudomycoides*, and *Bacillus pumilus* achieved a weight loss of 73.4% for PET powder (300 μm) in 18 days using the response surface methodology^26^. *Paenibacillus naphthalenovorans* PETKKU2 degraded 9.48% of PET microplastic in 35 days using the Box-Behnken design. The genome analyses suggested an alternative PET depolymerization mechanism indicating the role of lipases and carboxylesterases^27^. Although PET degradation under mesophilic conditions by *Ideonella sakaiensis* 201-F6 remains a benchmark^65^, our study demonstrates the efficiency of native dump yard soil bacteria such as *G. mysorens* ASR 14 under optimized bioprocess variables.

### Quantitative assessment of PET biodepolymerization by crude enzyme

**Fig. 6:**
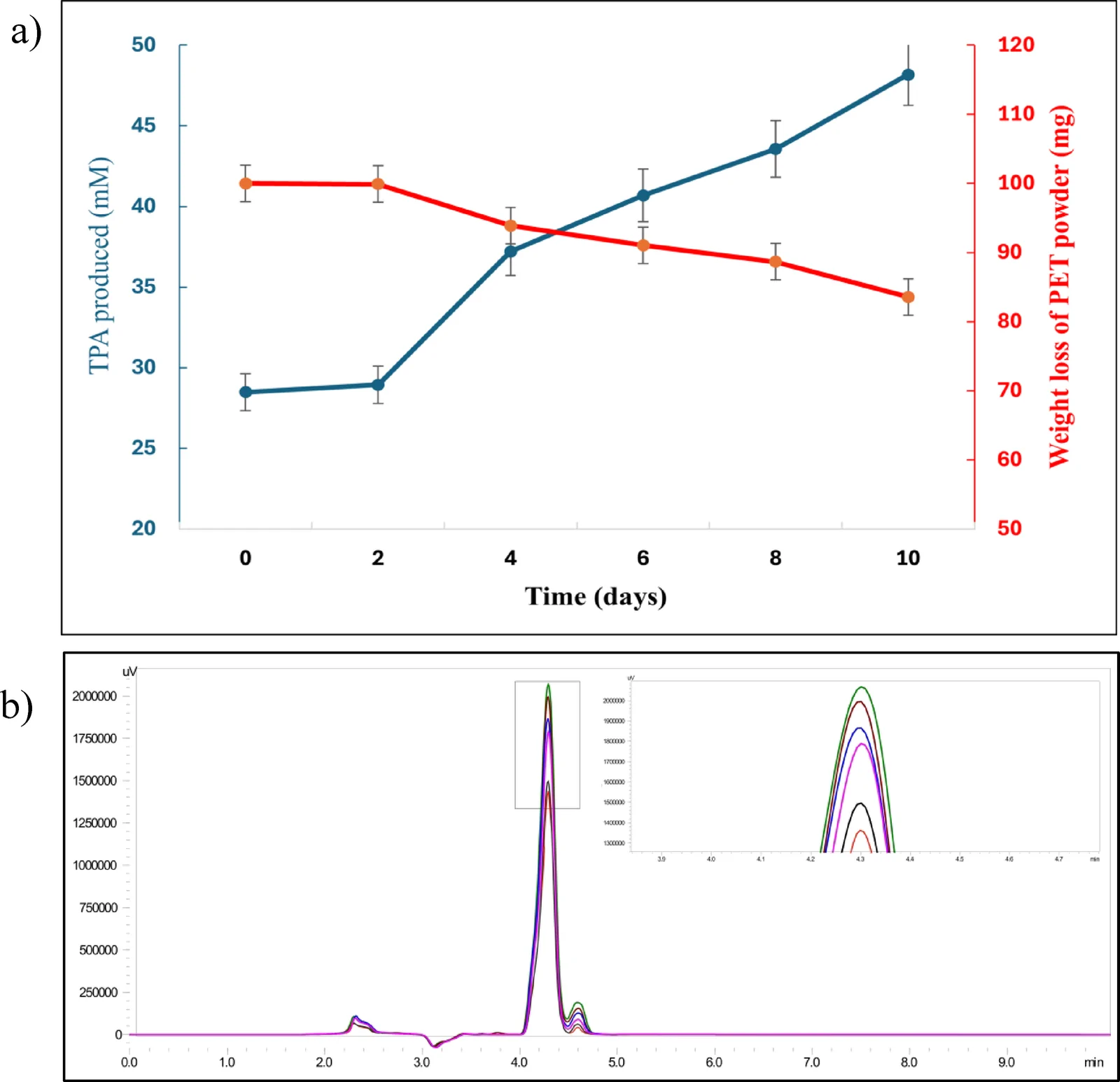
(**a**) TPA concentration and residual weight of PET powder over 10 d of crude enzyme treatment; (**b**) HPLC chromatogram showing production of TPA by PET hydrolysis using crude enzyme extract.

The biodepolymerization of PET powder using crude enzymes was systematically evaluated over a 10-day incubation period. The substrate achieved a weight loss of 16.45 mg and produced 48.2 mM TPA (Fig. 6a). The data showed a consistent increase in TPA accumulation parallel to the continual decrease in PET mass, as shown in Fig. 6b. This performance is much better than that of many reported enzyme systems, which typically showed 1–5% weight loss in several weeks^66,67^. In this study, 16.45% weight loss was observed after 10 d.

### Characterization of the degraded PET

#### Scanning electron microscopy and energy dispersive X-ray analysis

**Fig. 7:**
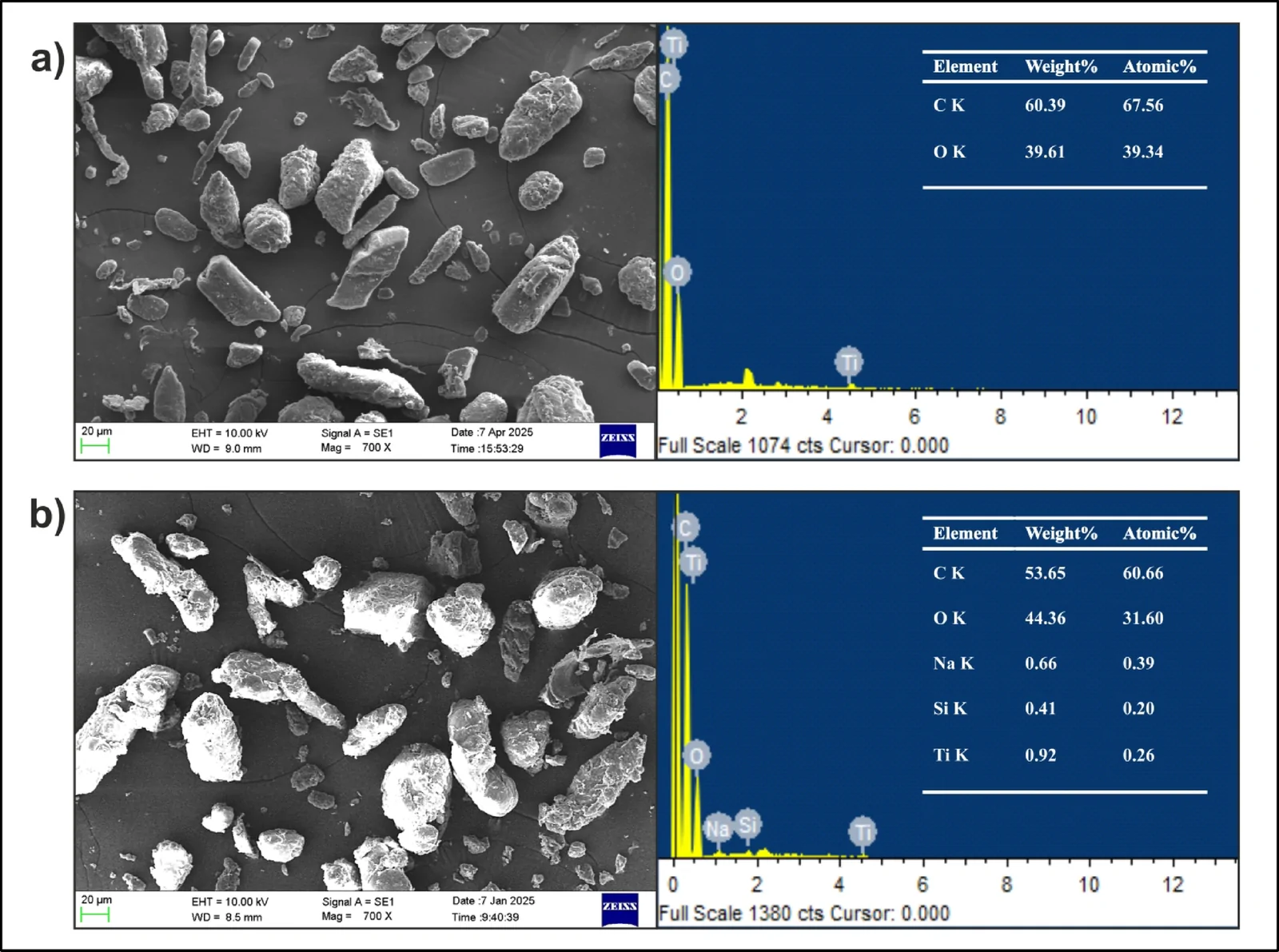
Scanning Electron Microscopy coupled with energy-dispersive X-ray spectroscopy of PET powder (a) Untreated PET powder (b) Biodegraded PET powder exhibiting surface erosion.

The PET powder before and after biodepolymerization, as observed by SEM, revealed distinct morphological variations. Before biodepolymerization, the powder showed a more defined and angular appearance, whereas post-biodepolymerization, it appeared porous and rougher in structure (Fig. 7). The SEM images are in accordance with the results of Dhaka et al., where the virgin PET film appeared smooth, and the biodegraded film appeared rough^42^. Similarly, SEM analysis by Gong et al. demonstrated the formation of pores and cracks on the polymer surface^68^. The EDS spectra showed a decrease in the carbon content from 60.39% to 53.65%, and additional peaks for sodium and silicon appeared post-biodepolymerization. This is in good agreement with the results reported by Skariyachan et al.^69^.

#### TPA by thin layer chromatography

**Fig. 8:**
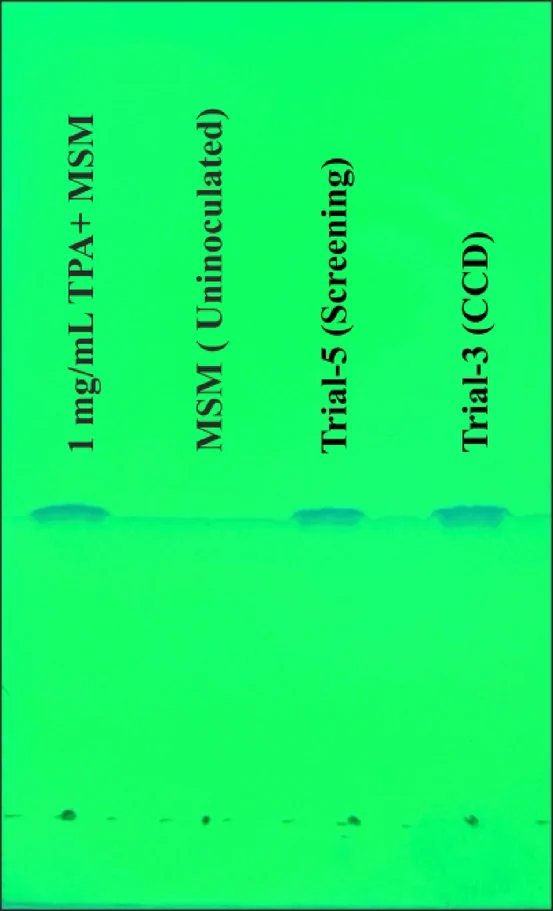
TLC analysis for detection of TPA from PET biodepolymerization.

Thin-layer chromatography was used as a qualitative analytical method to determine the presence of TPA resulting from PET biodepolymerization. This method is rapid and cost-effective for assessing bacterial depolymerization efficiency. The TPA spots were visualized using a UV lamp at 254 nm (Fig. 8). The presence of spots in Trial-5 and Trial-3 corresponding to the TPA standard (positive control) and their absence in the uninoculated MSM (negative control) confirmed the production of TPA by the isolated microorganism^70^. The acetonitrile-based solvent provides a simple and reliable approach for the qualitative detection of TPA, representing an improvement over previously reported acid-based solvents^71^.

#### Results from ATR- FTIR spectroscopic analysis

**Fig. 9:**
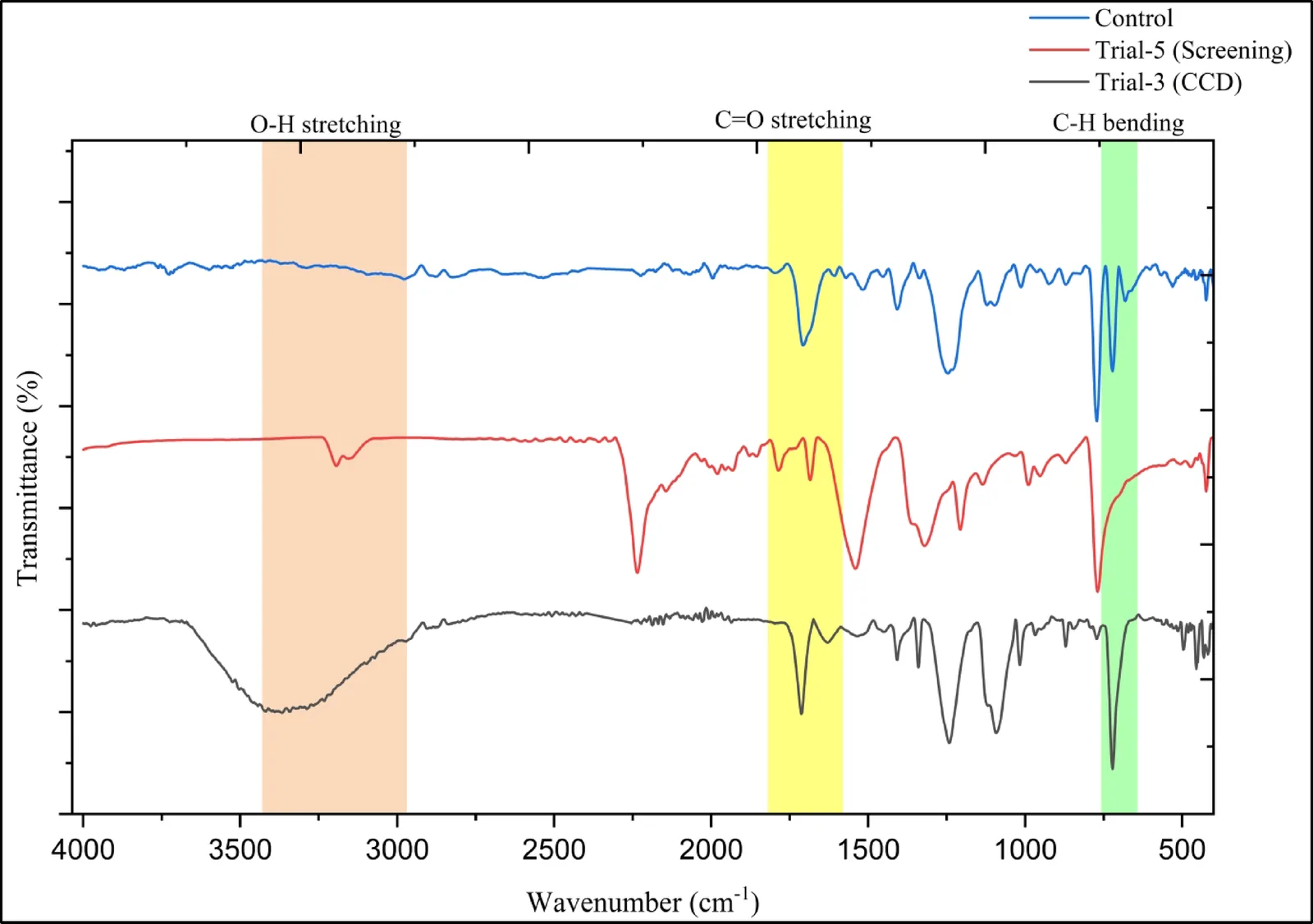
FTIR spectra comparing the control, Trial-5 (screening), and Trial-3 (CCD) samples, highlighting the key absorption bands.

ATR-FTIR analysis of PET biodepolymerization revealed changes in the characteristic peaks, demonstrating the involvement of microbial processes. The control spectra displayed the characteristic peak of native PET with an ester carbonyl stretch peak at approximately 1715 cm^− 1^, C-H bending at 725 cm^− 1^, and C-O-C stretching at 1250 cm^− 1^^72^. The FTIR spectrum of Trial-5 showed spectral changes, indicating early depolymerization. A reduction in the intensity of the carbonyl peak at approximately 1700 cm^− 1^ suggested partial hydrolysis of the ester bonds. Additionally, the emergence of the band in 3300–3500 cm^− 1^ region corresponding to the OH stretch indicated the formation of depolymerization end products, and the presence of a new peak in the 1600 cm^− 1^ region indicated the formation of acid or aldehyde intermediates. These changes are consistent with the findings of previous studies, in which similar spectral changes were observed during PET degradation^73^. Trial-3 of CCD exhibited the most profound changes in the FTIR spectrum compared to the control and Trial-5 of the screening design. An increase in the O-H peaks at approximately 3300 cm⁻¹ was observed, suggesting hydroxyl formation, a hallmark of PET biodepolymerization that aligns with the formation of TPA and EG^74^. The irregularities in this region, corresponding to the free O-H groups of alcohols and carboxylic acids, were also observed by Mismisuraya et al.^75^. The significant decrease in the peak at 1715 cm^− 1^ indicated C = O stretch, an ester bond hydrolysis, implying a reduction in chain integrity. Other peaks included 1410 cm^− 1^, indicating the presence of a benzyl ring. Vibrations of the ester group were detected at 1095.88 cm^− 1^. Peaks at 872 cm⁻¹ indicated C–H out-of-plane bending within the aromatic structure, and the peak at 493 cm^− 1^ indicated the out-of-plane vibration of = C-H in the benzene group. The decrease in the benzene ring peaks at 725 cm⁻¹ and 1470 cm⁻¹ indicated the breakdown of the aromatic ring of PET. The FTIR peaks of the control and biodegraded PET are shown in Fig. 9. The comparative analysis showed a progressive depolymerization trend from Trial-5 to Trial-3, marked by the disappearance of peaks related to ester linkages and the emergence of hydroxyl-associated peaks.

#### HPLC results

**Fig. 10:**
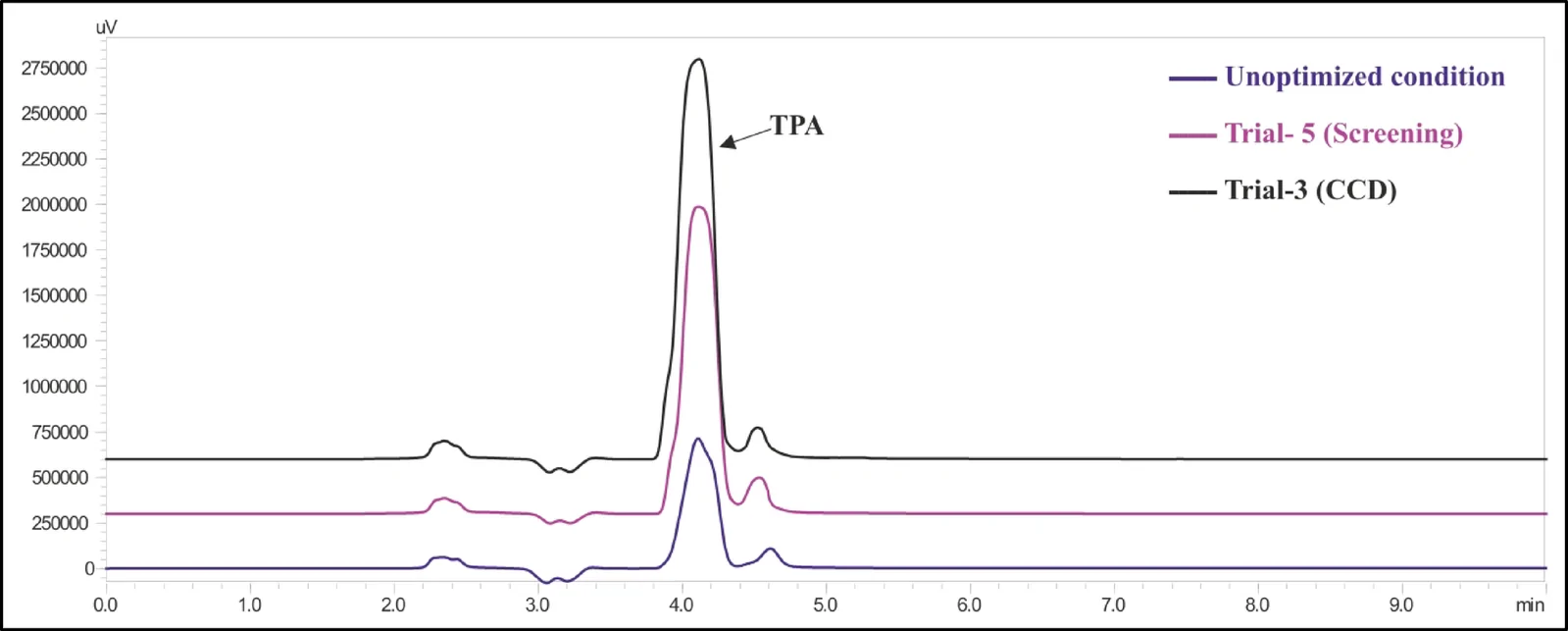
HPLC chromatograms showing the presence of TPA in the cell-free supernatant due to PET biodepolymerization.

HPLC analysis quantified the TPA released during PET biodepolymerization by *G. mysorens* ASR 14. The TPA standard exhibited a retention time of 4.31 min (Fig. 10). The concentration of TPA in the cell-free extracts was quantified using a calibration curve (Fig. S3). The calibration curve showed good linearity with R^2^ value of 0.9986. The calculated LOD and LOQ values were 0.065 µg/mL and 0.169 µg/mL, respectively. The concentrations of TPA produced in the cell-free extracts of all experimental trials of the screening of variables and CCD are summarized in Tables 2 and 4. Trial-5 of the screening phase and Trial-3 of CCD showed the highest percentage of PET biodepolymerization, producing 42.16 mM and 60.74 mM of TPA, respectively. This highlights the potential of bacteria to degrade PET under shake flask conditions. Genetically modified *Yarrowia lipolytica* yielded 124 mg/L of TPA after 96 h^76^. 129 mM of TPA was produced from *Humicola insolens* using post-consumer PET^77^. A recent study by Zeng et al.^78^ using *Bacillus safensis* YX8 reported TPA production of 2.9 × 10^− 3^ µM from PET nanoparticles. Compared to other studies, the ability of the bacteria to release 60.74 mM TPA without any genetic modification and enzyme enhancement signifies the noteworthy PET-degrading ability of this strain.

#### GC-MS results

**Fig. 11:**
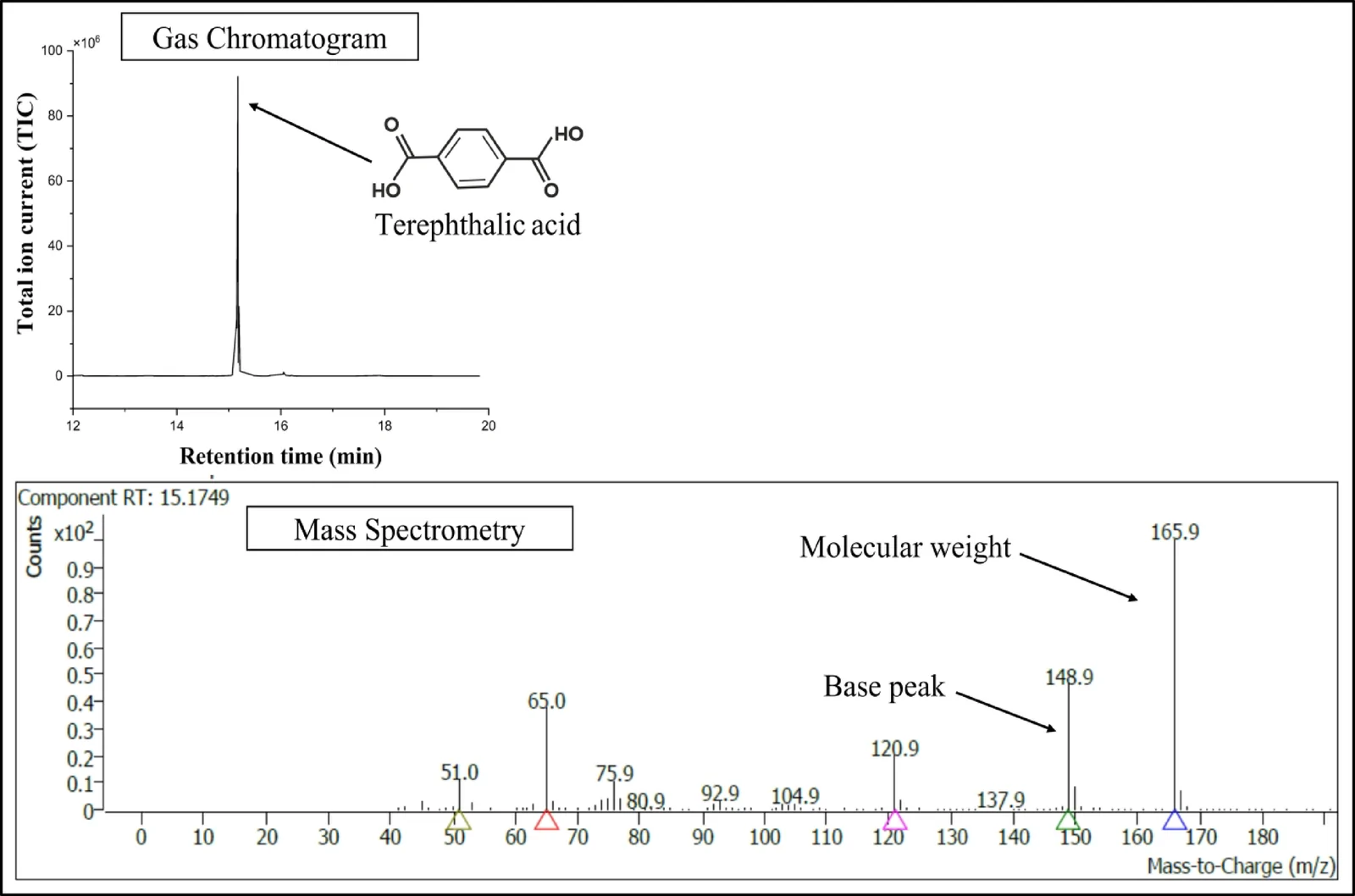
Gas chromatogram and mass spectrum of TPA detected in a cell-free extract with a retention time of 15.17 min showing a base peak at 148.9 m/z and molecular peak ion at m/z 165.9.

The cell-free extracts from the screening (Trial-5) and CCD (Trial-3) were subjected to GC-MS analysis, indicating the presence of key metabolites (Table S2) and TPA (Fig. 11). The detection of the TPA peak (15.17 min) suggested the aromatic ring cleavage of PET^40^. The EI spectrum showed a base peak at m/z 148.9 and a molecular peak at 165.9 (≈ M^+^ for C_8_H_6_O_4_), which matched the expected ions of TPA. The base peak at 148.9 m/z signifies the cleavage of an -OH/-COOH group from the carboxyl moiety. The fragmentation of TPA may further involve decarboxylation or decarbonylation, resulting in benzene carboxylate fragments^79^. The derivatization of cell-free extract detected metabolites such as 2,4-dihydroxybenzaldehyde and catechol derivatives such as 3-isopropyl-1,2-benzene diol and 4-propan-2-benzene-1,3-diol, which may further support depolymerization of the PET backbone^65^. No other significant PET depolymerization intermediates, such as MHET and BHET, were identified, representing a limitation of the present analytical characterization. The GC-MS results were in accordance with those of TLC and HPLC, validating the presence of TPA. This indicates that *G. mysorens* ASR 14 possesses PET-hydrolyzing capacity, metabolizes TPA to protocatechuic acid (PCA), and assimilates it^47^.

#### Raman analysis results

**Fig. 12:**
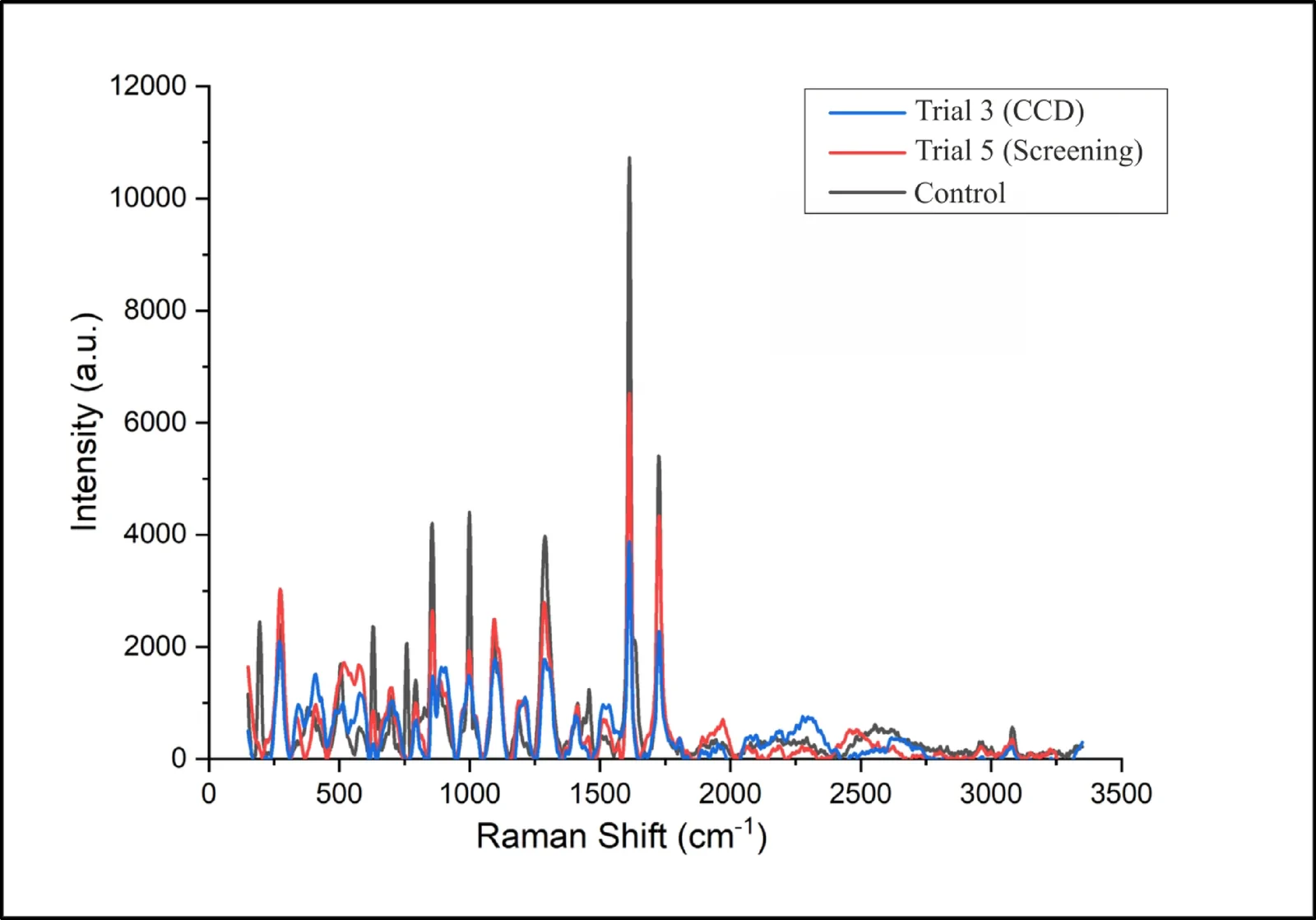
Raman spectra of PET powder: Control, Trial-5 (screening), and Trial-3 (CCD).

The Raman spectrum of pristine PET exhibited several distinct peaks characteristic of its molecular structure, primarily arising from ester linkages and aromatic moieties. The Raman spectra of the PET powder before and after biodepolymerization are shown in Fig. 12. A prominent peak was observed at approximately 1612 cm⁻¹, corresponding to the C = C stretching vibrations of the aromatic phenylene ring, which is a key structural component of the PET backbone^80^. Another strong and well-defined peak, located near 1725 cm⁻¹, is attributed to the C = O stretching vibrations of the ester functional groups, which are responsible for the chemical stability and mechanical strength of the polymer^81^. In addition, a notable band at 1457 cm⁻¹ arises from CH₂ bending or deformation vibrations, while the C–O–C asymmetric stretching vibrations of the ester linkage are evident at 1092 cm⁻¹ and 1113 cm⁻¹, reflecting the glycol subunit connectivity within the PET chain^82,83^. A sharp and intense peak centered at approximately 998 cm⁻¹ is characteristic of aromatic ring breathing modes, further confirming the presence of the benzene ring in the polymer backbone^84^. The spectral region between 870 cm⁻¹ and 880 cm⁻¹ corresponds to C–H out-of-plane bending vibrations associated with the aromatic ring, and a band appearing around 720–730 cm⁻¹ is typically assigned to CH₂ rocking or aromatic ring deformation. At the lower end of the spectrum, a band at 625 cm⁻¹ is indicative of benzene ring skeletal deformation, providing additional confirmation of the aromatic content of the polymer^85^.

In the high-frequency region, C–H stretching vibrations are observed, with a band near 2965 cm⁻¹ corresponding to aliphatic –CH₂– stretching and another at 3086 cm⁻¹ assigned to aromatic C–H stretching, which further validates the presence of both aliphatic and aromatic segments in the PET structure^82–84^. Notably, a decrease in the intensity of the carbonyl (C = O) stretching band around 1725 cm⁻¹, the aromatic C = C stretching band at 1612 cm⁻¹, and the aromatic ring breathing mode near 998 cm⁻¹ was observed. These reductions in spectral intensity are indicative of the cleavage of ester linkages and disruption of the aromatic framework, suggesting that the polymer underwent hydrolytic and possibly oxidative depolymerization processes^83^. Such spectral intensity losses serve as evidence of the polymer backbone breakdown, particularly at chemically susceptible sites such as the ester and aromatic units. Moreover, a comparative analysis between different experimental trials showed that Trial-3 (CCD) exhibited a greater reduction in peak intensities relative to Trial-5, suggesting a higher extent of biodepolymerization.

**Fig. 13:**
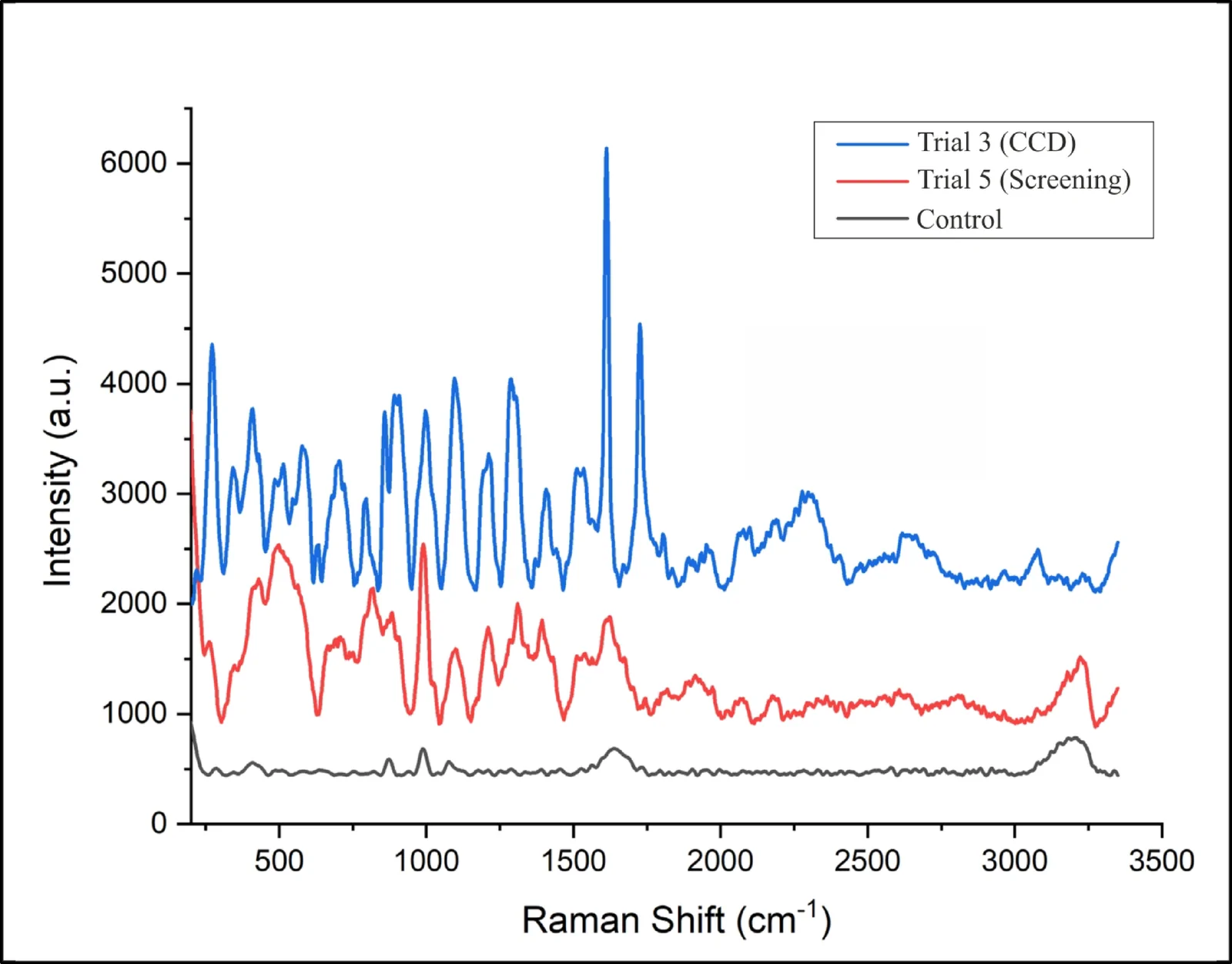
Raman spectra of cell-free extract of Control (MSM), Trial-5 (screening), and Trial-3 (CCD).

The Raman spectra of the cell-free extracts are shown in Fig. 13. An intense Raman band observed at 3205 cm⁻¹ in the control strongly suggested the presence of O–H stretching vibrations, typically associated with water, hydroxyl groups in medium constituents, or microbial byproducts^86^. The enhanced intensity of this band across the samples may also indicate the accumulation of hydroxyl-rich intermediates or polysaccharides during microbial activity. The next prominent band, appearing at 1638 cm⁻¹, is indicative of aromatic C = C stretching, which could originate from residual PET polymer or aromatic depolymerization products, such as TPA. This peak serves as a marker for aromatic integrity, and its variations across the samples suggest different extents of PET backbone depolymerization. The Raman band at 1078 cm⁻¹ is attributed to C–O stretching vibrations, potentially arising from ester bond cleavage in PET^84,87^. This band may also be influenced by extracellular polysaccharides produced by G.mysoresns ASR 14. A distinct band at 986 cm⁻¹ is characteristic of P–O stretching, associated primarily with phosphate salts in the mineral medium (Na₂HPO₄, KH₂PO₄)^88^. This band is typically stable and serves as a spectral reference for monitoring changes due to depolymerization processes.

A comparison of the Raman spectra of Trial-5 and Trial-3 reveals significant differences in the vibrational features. In Trial 3, a distinct peak at 1732 cm⁻¹ was observed, which was attributed to the C = O stretching vibrations of the ester functional groups present in the intact PET polymer. This peak was absent in Trial-5, suggesting that hydrolysis of the ester bonds occurred, leading to a higher degree of polymer breakdown. Additionally, Trial-3 displayed an intense peak at 1612 cm⁻¹, corresponding to the C = C stretching vibrations of the aromatic phenylene ring, a defining component of the PET backbone. In contrast, Trial-5 exhibited a comparatively weaker peak at 1622 cm⁻¹, which can be assigned to the C = C stretching of TPA, a primary depolymerization product of PET. This slight spectral shift, along with the reduction in peak intensity, supports the conclusion that aromatic structures remain in the supernatant but predominantly as low-molecular-weight products such as TPA, rather than as part of the intact polymer matrix.

#### NMR results

**Fig. 14:**
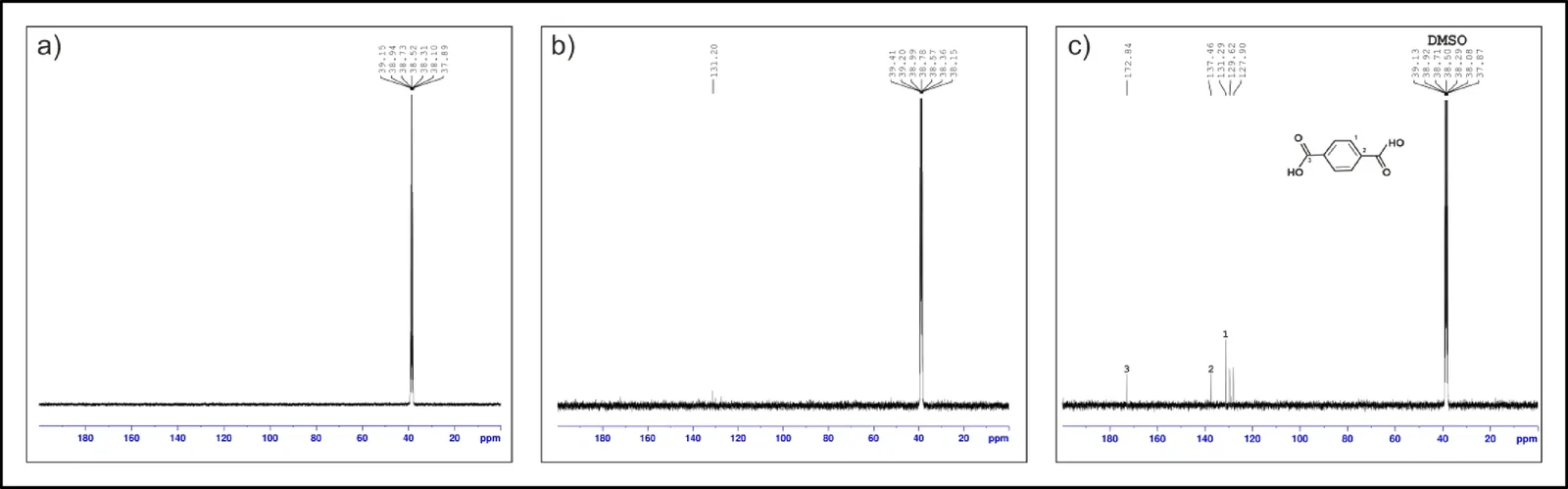
^13^C NMR **s**pectra of the cell-free extract showing characteristic peaks corresponding to TPA. (**a**) Control (**b**) Trial-5 (Screening) (**c**) Trial-3 (CCD).

NMR analysis of the PET cell-free extract provided insights into specific end products. Both ^1^H and ^13^C NMR spectra were recorded to identify the depolymerization products (Fig. 14). In the^13^ C NMR spectrum, the control showed a solvent (DMSO) peak at ~ 39 ppm and no significant signals in the aromatic or carbonyl regions. The increased noise at approximately 180 ppm showed the formation of carbonyl products. A new peak in the 130–135 ppm range in Trial-5 and Trial-3 indicated an aromatic carbon atom. The significant peak at ~ 167–172 ppm corresponds to a low-molecular-weight carboxylic acid. These results are consistent with previously reported data and commercially available TPA^89^. The increase in the intensity and number of peaks in Trial-3 indicated a greater accumulation of depolymerization products^90^. ^1^H NMR spectra recorded for the trials showed no significant changes in the chemical shift. A single sharp peak at 4.2 ppm was observed, signifying the presence of an O-H bond (Fig. S9). No aromatic peaks were observed in the 7–8 ppm region, indicating the absence of aldehydic or carboxylic regions, which is contrary to the findings reported by Roberts et al.^91^. While the GC-MS analysis identified trace-level intermediates of PET biodepolymerization, the corresponding signals were not observed in the ^1^H NMR spectrum due to relatively lower sensitivity of NMR in detecting trace metabolites in biological samples^92^.

#### XRD results

**Fig. 15:**
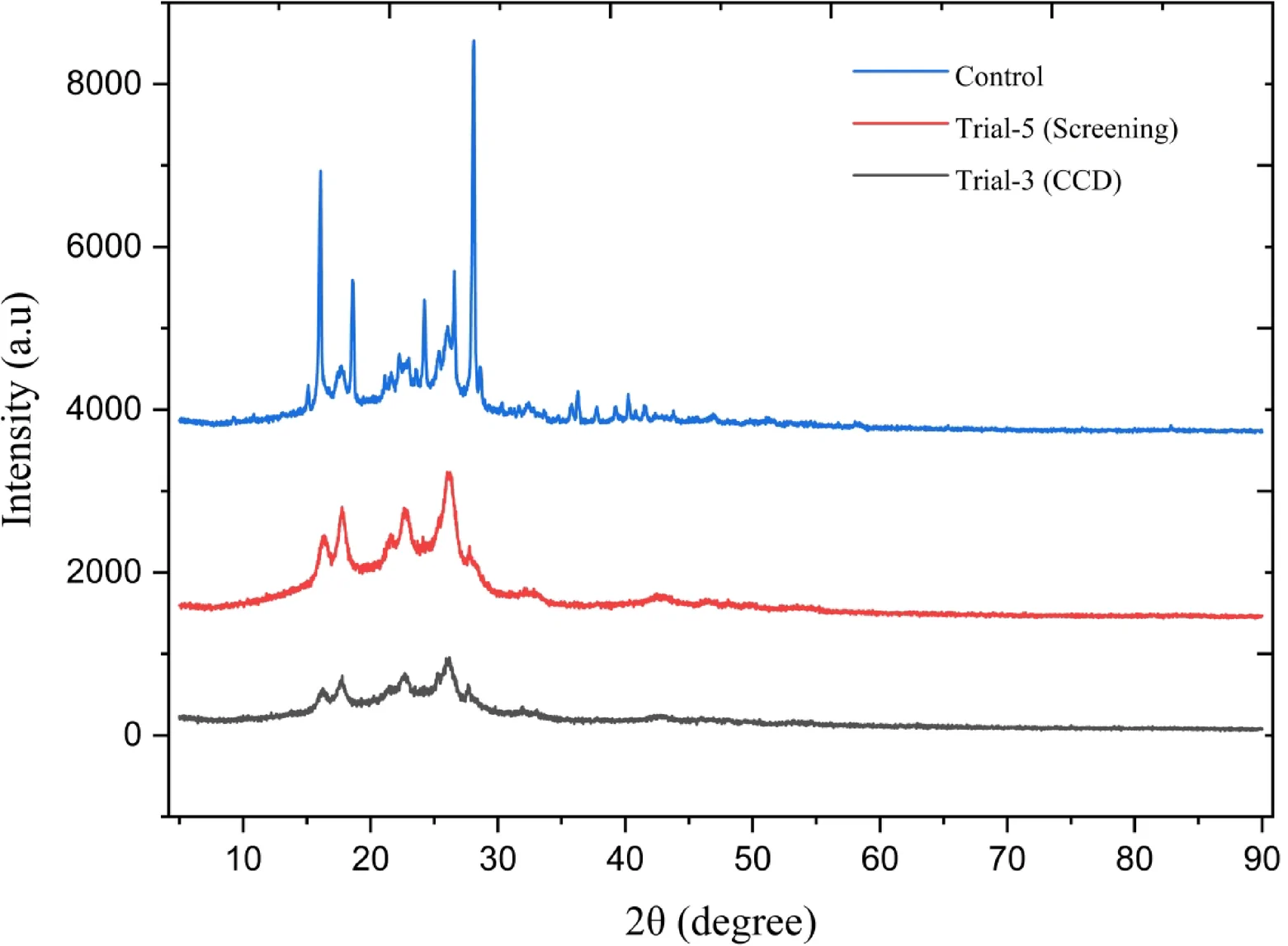
X-ray diffraction patterns of PET powder before and after biodepolymerization. A reduction in peak intensity and crystallinity was observed in Trial No-5 (Screening) and Trial No-3 (CCD).

The X-ray diffractogram of the control PET exhibited distinct peaks at 16.2°, 17.6°, 22.5°, and 25.8°, associated with the (010), (110), and (100) lattice planes, confirming semicrystalline domains^93^. Trial-5 exhibited a reduction in crystallinity, suggesting that the bacteria initiated depolymerization in the amorphous region. The maximum crystallinity loss was observed in Trial-3, suggesting enhanced depolymerization under CCD-optimized conditions (Fig. 15). A reduction in crystallinity was reported during the 90-day microbial degradation study on PET powder^64^. No new diffraction peaks appeared, confirming that the polymer remained in the same phase. The crystallinity index (CI) was calculated using the Segal method^94^. The CI reduced from 91.57% (control) to 79.08% (screening) to 20.18% (CCD), marking the loss of ordered crystallites with optimization, thereby enhancing hydrolysis^95^. The steady decrease in CI from Trial-5 to Trial-3 by *G. mysorens* ASR 14 indicated that the crystalline domains were also disrupted along with the amorphous region^96^.

## Conclusion

Under the experimental conditions set in this study, it was demonstrated that 27.6. % of the exposed PET was degraded within 30 days. Optimization of the process parameters significantly enhanced the depolymerization by 2.73 times, with the highest rate of 75.6% being obtained in 60 d. Enzymatic assays of esterase, lipase, and protease activities further promoted PET depolymerization. The exposed PET experienced marked surface erosion and a decrease in crystallinity due to biodepolymerization. This study provides the first comprehensive insight into the potential for PET biodepolymerization by *G. mysorens* ASR 14. This study advances a green strategy for plastic waste remediation by integrating whole-cell biocatalysts with process optimization. Overall, the findings have shown G. *mysorens* ASR 14 to be a promising candidate for sustainable, scalable, and eco-friendly bioremediation strategies for plastic waste. Limitations of the present study include the lack of comprehensive mass balance analysis, carbon dioxide evolution studies and total organic carbon (TOC) reduction assessment. Hence the biodegradation efficiency reported herein primarily reflects PET biodepolymerization rather than complete mineralization of the polymer. Furthermore the intermediates MHET and BHET were not detected during metabolite analysis under the experimental conditions in this study. Future studies may focus on metabolic pathways, mass balance ratios, enzyme characterization, and pilot-scale validation, all of which are crucial for translating these findings into practical environmental solutions. The high level of TPA produced can be used for biorefinery purposes by converting waste PET into other value-added products.

## Supplementary Information

Below is the link to the electronic supplementary material.

Supplementary Material 1

## Data Availability

Sequence data that support the findings of this study have been deposited in GenBank with accession number PQ813653. Additionally, data are provided within the manuscript and supplementary information files.
