# Supplementary material for: Systematic optimization and characterization of bacterial depolymerization of poly(ethylene terephthalate) plastics
Source: Sci Rep. 2026 Jun 20;16:28250. doi: 10.1038/s41598-026-58899-9 (PMC13558623; doi:10.1038/s41598-026-58899-9)
Supplement: Supplementary file 1 [file 41598_2026_58899_MOESM1_ESM.docx]

**Supplementary materials**

**Supplementary Figure Legends**

**Fig. S1.** Wavelength scan to determine the λ_max_ of Terephthalic acid in minimal salt media using a UV-Vis spectrophotometer. For terephthalic acid, the maximum absorbance was observed at 242 nm

**Fig. S2.** TPA standards (0.1mM to 1mM) in MSM and quantification at 242nm using UV-Vis spectrophotometry for calibration.

**Fig. S3.** A linear standard calibration curve of TPA in MSM analyzed using HPLC obtained by plotting TPA concentrations ranging from 0 to 500 μg/mL against peak area

**Fig. S4.** Partial 16S rRNA gene sequence of G.mysorens ASR14

**Fig. S5.** Biochemical characterization tests

**Fig. S6.** A Normal Probability plot representing the correlation between predicted and experimental percentage biodegradation in the customized screening design with a coefficient of determination (R^2^) of 0.90 and a statistically significant p-value of 0.0005.

**Fig. S7.** A Normal Probability plot representing the correlation between predicted and experimental percentage biodegradation from the central composite design model with a coefficient of determination (R^2^) of 0.92 and a statistically significant p-value of 0.0009.

**Fig. S8.** a) Residual versus predicted plot b) studentized residual plot for the CCD model

**Fig. S9.** ¹H NMR spectra of the cell-free extract showing only a single peak corresponding to the -OH bond of water, indicating the absence of other proton-containing compounds. a) Control b) Trial-5 (Screening) c) Trial-3 (CCD)

**Fig. S1.** Wavelength scan to determine the λ_max_ of Terephthalic acid in minimal salt media using a UV-Vis spectrophotometer. For terephthalic acid, the maximum absorbance was observed at 242 nm

**Fig. S2.** TPA standards (0.1mM to 1mM) in MSM and quantification at 242nm using UV-Vis spectrophotometry for calibration

**Fig. S3.** A linear standard calibration curve of TPA in MSM analyzed using HPLC obtained by plotting TPA concentrations ranging from 0 to 500 μg/mL against peak area.

**Fig.S4.** Partial 16S rRNA gene sequence of G.mysorens ASR 14

**Fig. S5.** Biochemical Characterization Tests: a) Methyl Red test; b) Voges-Proskauer test; c) Indole Acetic Acid (IAA) test; d) Triple Sugar Iron (TSI) test; e) Starch hydrolysis test

**Fig. S6.** A Normal Probability plot representing the correlation between predicted and experimental percentage biodegradation in the customized screening design with a coefficient of determination (R^2^) of 0.90 and a statistically significant p-value of 0.0005.

**Fig. S7.** A Normal Probability plot representing the correlation between predicted and experimental percentage biodegradation from the central composite design model with a coefficient of determination (R^2^) of 0.92 and a statistically significant p-value of 0.0009.

a)

**Fig.S8.** a) Residual versus predicted plot b) studentized residual plot for the CCD model

**Fig. S9.** ¹H NMR spectra of the cell-free extract showing only a single peak corresponding to -OH bond of water, indicating the absence of other proton-containing compounds. a) Control b) Trial-5 (Screening) c) Trial-3 (CCD)

**Supplementary Table Legends**

**Table S1** Parameter estimates of significant variables

**Table S2** GC-MS profiling of metabolites produced during microbial degradation of PET

| **Term** | **Scaled Estimate** |  | **Std Error** | **Prob>\|t\|** |
| --- | --- | --- | --- | --- |
| Intercept | 18.20684 |  | 0.78408 | <.0001* |
| X2[L1] Yeast Extract | 3.9484871 |  | 1.313746 | 0.0397* |
| X2[L2] Peptone | 7.5827696 |  | 1.462018 | 0.0066* |
| X2[L3] Casein | -3.918478 |  | 1.399772 | 0.0488* |
| X2[L4] Soyabean meal | -7.612779 |  | 1.415998 | 0.0058* |
| X3[L1] Tween 20 | -0.707699 |  | 1.191362 | 0.5845 |
| X3[L2] Triton X-100 | 2.7074373 |  | 1.126939 | 0.0542 |
| X3[L3] SDS | -1.999738 |  | 1.144807 | 0.1556 |
| X4[L1] MSM | 3.9235912 |  | 0.807149 | 0.0083* |
| X4[L2] BHB | -3.923591 |  | 0.807149 | 0.0083* |
| X5[L1] No Amino acid | 1.6567228 |  | 1.410743 | 0.3054 |
| X5[L2] Leucine | -4.354154 |  | 1.427854 | 0.0380* |
| X5[L3] Glutamine | -1.369066 |  | 1.431442 | 0.3930 |
| X5[L4] Cysteine | 4.0664967 |  | 1.426354 | 0.0464* |
| X6[L1] 45 | 0.4402156 |  | 1.16671 | 0.7251 |
| X6[L2] 90 | 0.1412997 |  | 1.057489 | 0.9002 |
| X6[L3] 150 | -0.581515 |  | 1.165624 | 0.6440 |
| X7 (5% PET) | 4.4956283 |  | 0.800125 | 0.0049* |
| X8 (inoculum) | -0.599599 |  | 0.823119 | 0.5067 |
| X9 (200 rpm) | 2.771449 |  | 0.810086 | 0.0268* |
| X10 (pH) | 1.1875613 |  | 0.817553 | 0.2200 |

**Table S1** Parameter estimates of significant variables

**Table S2** GC-MS profiling of metabolites produced during microbial degradation of PET

| **Library molecular Weight (MW)** | **Compound Name** | **Retention Time (min)** | **Match Factor (>70 %)** |
| --- | --- | --- | --- |
| 152.084 | 4-(Propan-2-yl)benzene-1,3-diol | 8.819 | 70.033 |
| 166.027 | Terephthalic acid | 14.452 | 84.903 |
| 152.084 | 3-Isopropyl-1,2-benzenediol | 8.832 | 70.927 |
| 296.126 | 2,4-Dihydroxy-3-methylbenzaldehyde | 7.488 | 80.093 |
